# The azole-resistance phenotype of a *Nakaseomyces glabratus* clinical strain encoding a wild-type *PDR1* allele involves the efflux pumps Aus1 and Pdh1 and Cyb5, an alternative reductase required for ergosterol biosynthesis

**DOI:** 10.1128/spectrum.03344-25

**Published:** 2026-03-27

**Authors:** Maria Joana Pinheiro, Josie E. Parker, Maria T. Ferreira, Fábio Fernandes, Nuno Pereira Mira

**Affiliations:** 1 Instituto Superior Técnico–Department of Bioengineering, iBB, Institute for Bioengineering and Biosciences, Universidade de Lisboa 72971 , Lisboa, Portugal; 2 Associate Laboratory i4HB—Institute for Health and Bioeconomy at Instituto Superior Técnico, Universidade de Lisboa, Lisboa, Portugal; 3 Molecular Biosciences Division, School of Biosciences, Cardiff University 2112, Cardiff, Wales, United Kingdom; University of Guelph College of Biological Science, Guelph, Ontario, Canada

**Keywords:** azole-resistant *Nakaseomyces glabratus* strains, wild-type Pdr1 and Pdr1 gain-of-function mutants, azole resistance, ergosterol biosynthesis

## Abstract

Azole resistance among *Candida* yeasts is problematic as it increases therapeutic failure, resulting in prolonged hospital stays and worse clinical prognosis. Clinical strains of the *N. glabratus* species (formerly classified as *Candida glabrata*) acquire resistance to azoles mainly by acquiring gain-of-function (GOF) mutations in the regulator Pdr1. However, a small percentage of resistant strains without such modifications have been described. This work focuses on one of such azole-resistant strains encoding a “wild-type” (non-GOF) *PDR1* allele, ISTB218. We found that the azole-resistance phenotype in this strain involves: (i) the expression of the multi-drug resistance transporters Pdh1 and Aus1, whose deletion abolished the fluconazole and voriconazole resistance; (ii) an Aus1-mediated increase in plasma membrane fluidity, especially under fluconazole stress; and (iii) the ability to sustain higher pools of ergosterol and lower accumulation of methylated sterols under fluconazole stress. This last trait was linked to the activity of the *CYB5* gene, an alternative reductase that is overexpressed in ISTB218 cells and that we found, for the first time, to be required for 14-demethylation of lanosterol and 22-desaturation of episterol. Thus, Cyb5 is added to the panoply of enzymes involved in ergosterol biosynthesis in *N. glabratus*. Altogether, our results advance knowledge on the biosynthesis of ergosterol and the mechanisms of resistance to azoles independent of Pdr1, thus opening the portfolio of possible therapeutic targets to be used in the advance of new anti-*Candida* therapies.

**IMPORTANCE:**

The emergence of resistance to azoles among strains of *Nakaseomyces glabratus* is a major cause of concern, considering the importance of this yeast in causing human infections. In this work, we disclose a set of genes that mediate the azole-resistance phenotype of a resistant clinical strain, with one of these players (Cyb5) being also implicated in biosynthesis of ergosterol for the first time. With this, we increase a set of possible therapeutic targets that can be used in future studies to develop new antifungals.

## INTRODUCTION

The incidence of invasive fungal infections has risen significantly in the recent decades, resulting in 3.8 million deaths estimated worldwide yearly (1). Species of the *Candida* genus are among the more relevant agents of fungal infections, causing deadly disseminated infections that can result in colonization of solid organs, such as the brain, the kidney, or the heart (2). While such infections occur essentially in immunocompromised individuals, mucosal infections (e.g., in the oral or urogenital tracts) are frequent in immunocompetent individuals (2). Acknowledging the burden posed by candidiasis, the World Health Organization recently placed several *Candida* species in the “high” and “critical” priority list of pathogens, aiming to raise awareness for the importance of research focused on these species (3). Invasive and mucosal candidiasis are typically caused by *Candida albicans*, but infections caused by *N. glabratus* (formerly classified as *Candida glabrata*) are increasingly frequent, especially in Europe and North America (1, 2, 4). *N. glabratus*’s very high tolerance to environmental stress (much higher than that of *C. albicans*) is at the center of the success of this species as a pathogen (2, 5). Among other consequences, this high stress tolerance is reflected in reduced susceptibility to azoles, lower than the one exhibited by almost all other human-infecting *Candida* species (2, 5, 6). *N. glabratus* also acquires resistance to azoles very rapidly *in vitro*, possibly due to its extreme genomic plasticity, although the frequency of isolation of resistant clinical strains from this species (ranging from 3% to 46%, depending on the geography) is similar to the one observed for the other *Candida* species (7–10). This “innate” tolerance to azoles, along with the fast acquisition of resistance to azoles, makes infections caused by *N. glabratus* worrisome in the clinical setting due to increased therapeutic failure and worsened patient prognosis (11, 12).

Azoles halt fungal growth by disrupting ergosterol biosynthesis through the inhibition of the sterol 14α-demethylase Erg11. Such inhibition causes the accumulation of methylated sterols that integrate into the membrane, resulting in compromised functionality and integrity, although the exact mechanism by which this happens is still elusive (2, 13). Almost all azole-resistant *N. glabratus* clinical strains encode *ERG11* alleles without detectable changes in coding sequence or expression, compared to the alleles harbored by azole-susceptible strains, in contrast with what is observed in strains of other azole-resistant *Candida* species, where such modifications are frequent (2, 6). In *N. glabratus*, the predominant mechanism described in azole-resistant clinical strains is the occurrence of gain-of-function (GOF) mutations in the coding sequence of the transcription regulator Pdr1 (2, 6, 14). Although the way by which these GOF mutations affect the activity of Pdr1 is unclear, the enhanced expression of the multidrug efflux pump *CDR1* is a common response to these azole-resistant *N. glabratus* strains (6, 13, 15). As a result of *CDR1* overexpression, strains encoding Pdr1 GOF mutants avoid internal accumulation of azoles, reducing their toxic effects (2, 6, 15, 16).

Despite the central role played by Pdr1-dependent responses in azole resistance in *N. glabratus*, a few clinical strains that encode *PDR1* “wild-type” alleles (that is, alleles identical to those encoded by azole susceptible strains) have been identified, including ISTB218, which is at the center of this work (13, 14, 17–19). These observations suggest that resistance mechanisms independent of Pdr1 also contribute to *N. glabratus* resistance to azoles *in vivo*. Such a hypothesis is consistent with the results of large-scale phenotypic surveys, which revealed many genes conferring protection to azoles that are not under the control of the Pdr network (20, 21). The mitochondrial localization of fluconazole, detected by live-cell imaging in azole-exposed *Candida* (22), contrasts with Erg11 localization in the endoplasmic reticulum and suggests an alternative intracellular dynamics of azoles, as well as additional targets. Little work has been performed with these azole-resistant “*PDR1^wt^* strains,” and therefore, those Pdr1-independent responses remain largely uncharacterized, especially in the background of clinical isolates.

The present study focuses on the *N. glabratus* azole-resistant clinical strain ISTB218, which encodes a *PDR1* allele identical to the one encoded by azole-susceptible clinical strains, including the reference strain CBS138 and the clinical isolate ISTA29. ISTA29 was used in this study (and in prior ones) as an example of a clinical strain that, like CBS138, exhibits an azole-susceptible phenotype (see Fig. S1 for more information on genetic relatedness of the ISTB218 and ISTA29 strains) (13). Combining OMICS with detailed molecular analysis, we hypothesized that the azole-resistance phenotype of the ISTB218 strain could result from a higher level of expression of several genes described to increase protection to fluconazole in the background of laboratory strains (13). Herein, we confirm that the expression of the ABC transporters Pdh1 and Aus1, two of the genes found to be more actively transcribed in ISTB218 than in CBS138, is required for the azole-resistance phenotype of the strain. Based on the much higher number of vesicles enriched in ergosterol (detectable upon cell labeling with filipin) observed in ISTB218 than in CBS138 (or ISTA29), we proposed that ergosterol metabolism could be altered in this azole-resistant strain (13). Herein, we gather evidence confirming that, as the filipin labeling suggested, ISTB218 cells do harbor higher pools of ergosterol and lower pools of methylated sterols when under fluconazole stress—a trait that we could link to the activity of Cyb5 reductase. Using several approaches we show that Cyb5 can be added to the set of enzymes involved in ergosterol metabolism in *N. glabratus*, expanding current knowledge about the key players involved in this pathway and, in turn, broadening the body of therapeutic targets for the development of anti-*Candida* drugs.

## RESULTS

### The expression of the ABC transporters *PDH1* and *AUS1* contributes to azole resistance and plasma membrane properties of *N. glabratus* ISTB218

**Fig 1:**
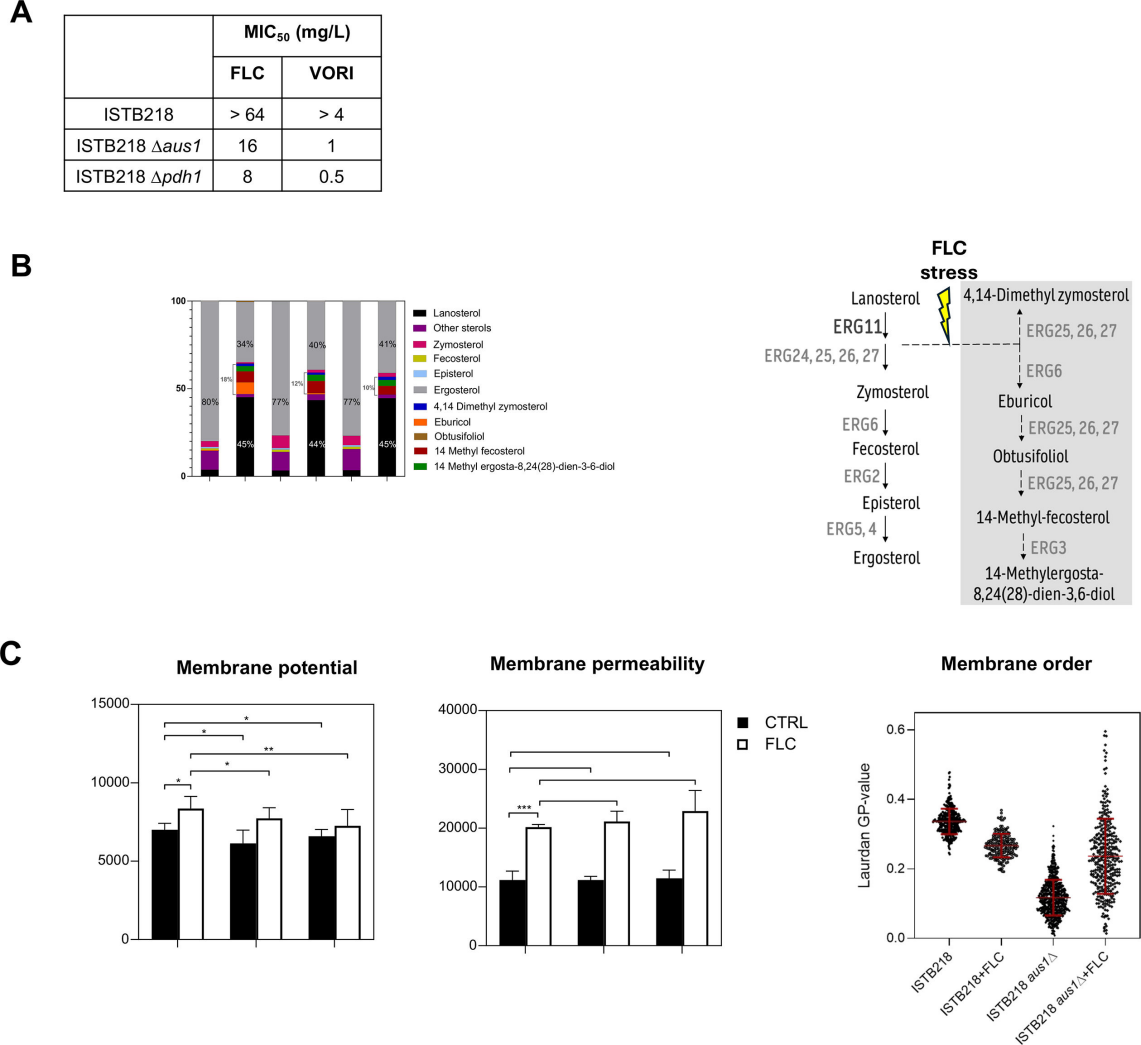
Effect of the expression of *CgPDH1* and *CgAUS1* genes on tolerance to azoles and on the properties of the plasma membrane of *C. glabrata* ISTB218 isolate. (**A**) Minimum inhibitory concentrations (MIC₅₀) of fluconazole and voriconazole of ISTB218, ISTB218 *aus1*Δ, and ISTB218 *pdh1*Δ strains, as quantified using the EUCAST-recommended standardized assay. In Fig. S1, the OD of the cultures obtained in the presence or absence of fluconazole and that resulted in the MICs herein presented are shown. The assays were performed in triplicate that gave, essentially, similar results. (**B**) Abundance of sterols in whole-cells of *C. glabrata* ISTB218, ISTB218_Δ*aus1,* and ISTB218_Δ*pdh1* cultivated, for 24 h, in the presence or absence of 32 mg/L fluconazole. The bars represent the relative proportion (% of the total) of each sterol, shown as the mean obtained in three independent biological replicates. The quantitative data used to build this graph are shown in Fig. S2. On the left of this panel, a simplified scheme of the ergosterol biosynthetic pathway in *C. glabrata* is shown, with the methylated sterols that accumulate under fluconazole stress upon Erg11 inhibition highlighted in gray. (**C**) Biophysical properties (order, electrochemical potential, and permeability) of the plasma membrane of ISTB218, ISTB218 *aus1*Δ*,* and ISTB218 *pdh1*Δ cells cultivated for 24 h in the presence or absence of fluconazole. Membrane order was estimated based on the calculation of GP values of the fluorescent probe laurdan, estimated in more than 100 individual cells, as detailed in Materials and Methods. The electrochemical potential was determined upon cell labeling with DiSC₃(5) (3,3′-Dipropylthiadicarbocyanine iodide), while membrane permeability was estimated upon cell labeling with rhodamine B. All the data represented are means ± SD of three independent biological replicates. For the statistical analysis, two-way ANOVA followed by Tukey’s post-hoc test was used. *P-value below 0.1; **P-value below 0.01; ***P-value below 0.001.

The *N. glabratus* ISTB218 strain is resistant to fluconazole (MIC_50_ > 64 mg/L) and voriconazole (MIC_50_ 8 mg/L), but this phenotype does not result from these cells encoding a gain-of-function *PDR1* allele (13). Transcriptomic analysis revealed that the expression of the ABC transporters *AUS1* and *PDH1* is higher in ISTB218 than in the azole-susceptible strain CBS138, 11- and 2-fold, respectively (13). Since *AUS1* and *PDH1* genes contribute to fluconazole tolerance in the background of laboratory strains (16, 23), we hypothesized that the same could also be true for ISTB218. Consequently, we deleted *AUS1* and *PDH1* from the ISTB218 genome, resulting in the abolishment of the resistance phenotype of the strain to fluconazole and voriconazole, with the deletion of *PDH1* having a stronger effect (Fig. 1A and Fig. S2). Afterward, we compared the sterol content of ISTB218, ISTB218_Δ*aus1*, and ISTB218_Δ*pdh1* whole cells cultivated for 24 h in RPMI medium, either or not exposed to fluconazole (32 mg/L). This was motivated by the demonstrated role of Aus1 as a sterol importer (23, 24). Consistent with what was expected and reported for other *N. glabratus* strains, exposure of ISTB218 (and also of the derived mutants) to fluconazole (32 mg/L) led to the accumulation of methylated sterols and of lanosterol (Fig. 1B and Fig. S3) (25). The abundance of the different sterols was, in general, identical in ISTB218 and in the two mutants, both in the presence and absence of fluconazole, with the only noticeable difference being an azole-induced accumulation of eburicol in ISTB218 (~6%), which was not observed in the Δ*aus1* or Δ*pdh1* mutants (Fig. 1B and Fig. S2). These findings support the idea that the protective effect exerted by Pdh1 and Aus1 against fluconazole is not related to a role in modulating sterol abundance. We then examined whether those two ABC transporters could impact plasma membrane order, permeability, and potential, considering prior demonstration that ABC-MDR transporters influence these parameters in other yeasts with consequences in their tolerance to different drugs (24, 26, 27). To estimate those parameters, we labeled cells from the three strains (ISTB218, ISTB218_Δ*aus1*, and ISTB218_Δ*pdh1*) with three fluorescent probes: DiSC_3_, to assess the membrane polarization and, indirectly, the electrochemical potential; rhodamine B, to assess membrane permeability; and laurdan, to assess plasma membrane order (28–30). Our exploration of these probes leveraged their reported success in accurately monitoring these key physiological parameters, both *in vivo* and *in vitro* . Importantly, we have monitored these parameters in the same experimental conditions that had been used to quantify the MIC50 of the strains to fluconazole, thus allowing us to establish meaningful correlations with the azole-resistance phenotype. In the absence of fluconazole, fluorescence of DiSC_3_-labeled ISTB218_Δ*aus1* and ISTB218_Δ*pdh1* cells was slightly below the one of ISTB218 (Fig. 1, on the right). DiSC_3_ accumulates close to the membrane in polarized cells, giving rise to low fluorescence values due to self-quenching (31). When a reduction in membrane potential is observed (in other words, when membrane depolarization is observed), DiSC_3_ is released to the cytosol, reducing self-quenching and increasing fluorescence (31). Thus, the higher fluorescence observed in the two mutant populations reflects a less polarized plasma membrane (or, in other words, a lower plasma membrane potential). ISTB218_Δ*aus1* and ISTB218_Δ*pdh1* cells also exhibited mildly higher values of rhodamine B fluorescence (Fig. 1C, in the middle), which can reflect a higher permeability of their membrane and a lower capacity to efflux the drug, which is consistent with the prior demonstration of rhodamine as a substrate of Pdh1 (32). The most significant difference detected between ISTB218 and the two mutants was the prominently low generalized polarization (GP) value estimated for laurdan in the Δ*aus1* population (Fig. 1C, on the left). Laurdan fluorescence emission spectrum depends on lipid packing in such a way that, in ordered membranes, the spectrum of the probe is shifted to lower wavelengths relative to what is observed in more disordered lipid membranes. This spectral shift and the consequent packing of the membrane are quantified in the GP factor, which is obtained from the ratio between the fluorescence intensity of two spectral channels in the laurdan emission spectrum (29). Thus, the marked decrease in GP registered in the Δ*aus1* mutant population reflects a clearly less ordered membrane, even in the absence of stress. Exposure to fluconazole led to reduced GP values for laurdan, reflecting increased membrane fluidity, a response that was also observed in *C. albicans* and *S. cerevisiae* stressed with fluconazole (33, 34). Differently, in the Δ*aus1* mutant population, the fluidity levels of the membrane increased upon fluconazole stress (Fig. 1C). Altogether, the results obtained sustain the idea that Aus1 (but not Pdh1) is involved in maintaining the fluidity of the plasma membrane of ISTB218 cells, especially under fluconazole stress.

### The fluconazole-induced inhibition of Erg11 and the alterations it causes in the biophysical properties of the plasma membrane are less evident in ISTB218 than in the azole-susceptible *N. glabratus* strains ISTA29 and CBS138

**Fig 2:**
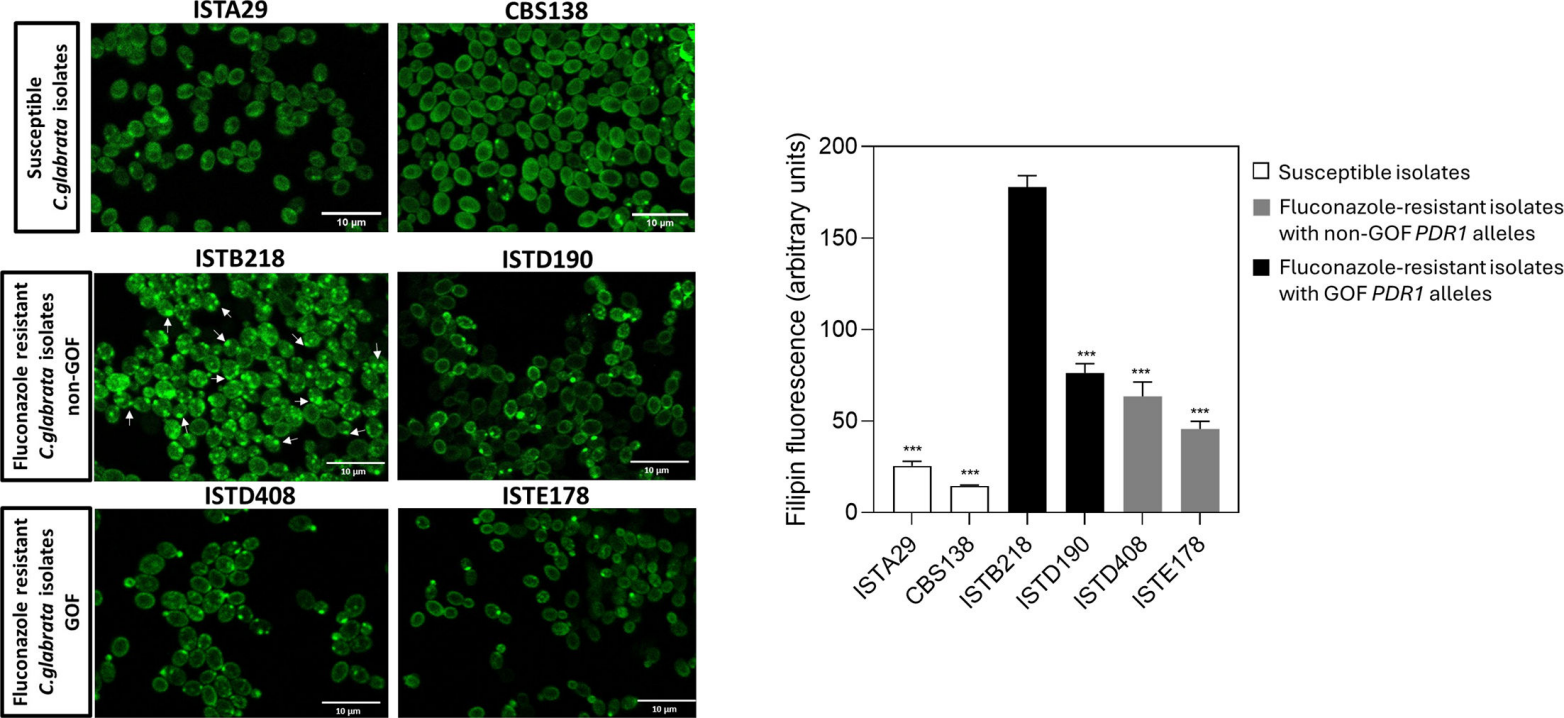
Filipin staining of *C. glabrata* clinical isolates susceptible or resistant to fluconazole. *C. glabrata* clinical isolates susceptible (ISTA29, CBS138) or resistant to fluconazole (ISTB218, ISTD190, STD408, ISTE178) were labeled with the ergosterol-binding dye filipin, during growth in RPMI medium and following the experimental setting described in Materials and Methods. Scale bar = 10 µm. ***P-value below 0.001.

**Fig 3:**
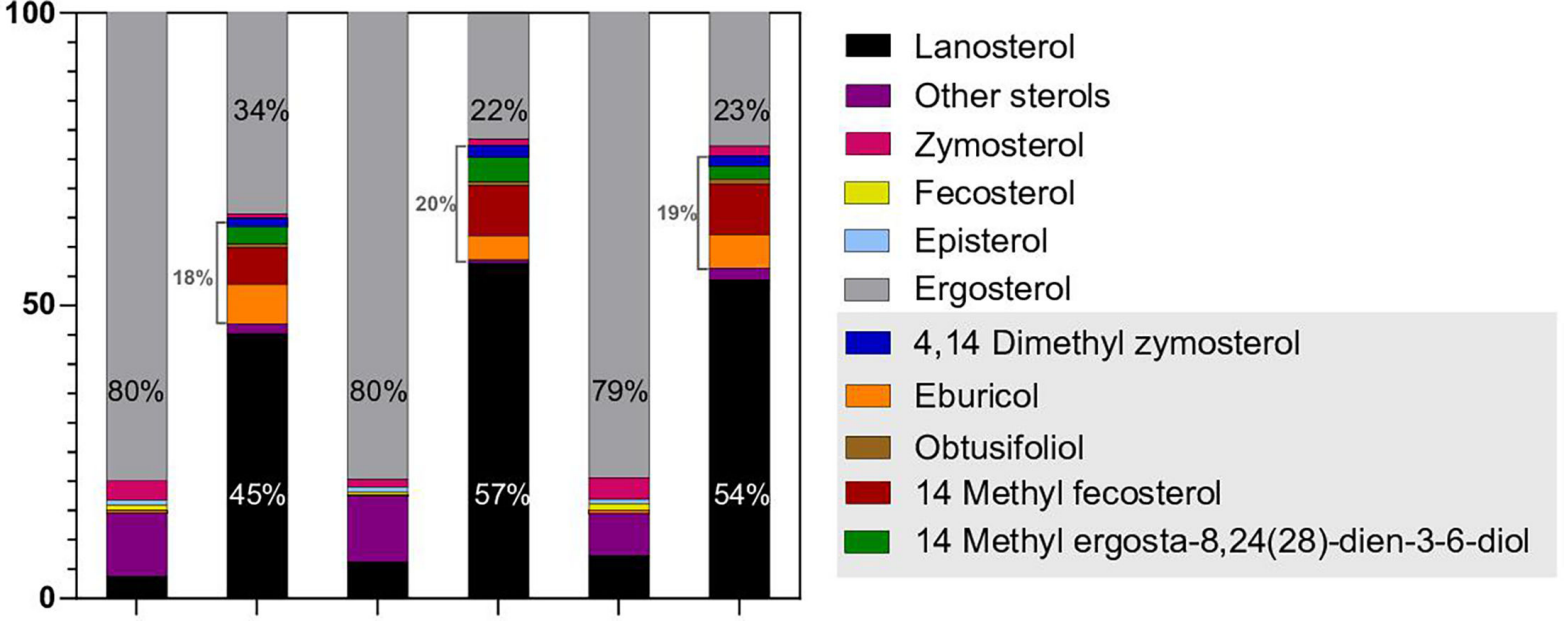
Sterol profile of whole *C. glabrata* cells from isolates ISTB218 (azole-resistant), ISTA29, and CBS138 (azole-susceptible) during cultivation for 24 h in the presence or absence of fluconazole. Using the experimental setup described in Materials and Methods, cells from the different isolates cultivated for 24 h in RPMI either supplemented with or without 32 mg/L of fluconazole were harvested, and their lipid fraction was recovered. The figure shows the abundance of different sterols collected in those lipid fractions (in % of the total), shown as the mean of three independent biological replicates. The total of methylated sterols, as well as the percentage of lanosterol and ergosterol obtained for the different isolates in the different conditions, is highlighted. Percentages are also shown for ergosterol and lanosterol. The quantitative data used to build this graph are shown in Fig. S5.

The formation at high extent of “ergosterol-enriched vesicles” (detectable upon labeling with filipin) in the cytosol of *N. glabratus* ISTB218 cells suggested that this strain could have alterations in ergosterol synthesis (13). We used filipin to label 11 other azole-resistant *N. glabratus* strains of our collection (including strains encoding wild-type or GOF Pdr1 alleles) and the azole-susceptible strains CBS138 and ISTA29. The results obtained (shown in Fig. 2 and in Fig. S4) confirmed the formation of the “ergosterol-enriched vesicles” in all the examined strains (including in the two azole-susceptible strains), but their number was clearly higher in ISTB218 (as we have previously documented), resulting in this strain producing the highest levels of filipin fluorescence (Fig. 2 and in Fig. S4). Notably, the formation of similar cytosolic “ergosterol-enriched” structures labeled by filipin was reported in *S. cerevisiae* (23), confirming that the observations made herein and in our previous study are not an artifact. The higher fluorescence values produced by filipin labeling in ISTB218 cells were suggestive of higher internal pools of ergosterol, which was confirmed by LC-MS-specific quantification (Fig. 3 and Fig. S5) (13). The higher ergosterol pools were the most notable difference between the sterol pools of ISTB218 and those of the susceptible strains ISTA29/CBS138 (Fig. 3). Another point of remark was the lower accumulation of methylated sterols and of lanosterol in fluconazole-challenged ISTB218 cells (Fig. 3 and Fig. S5).

**Fig 4:**
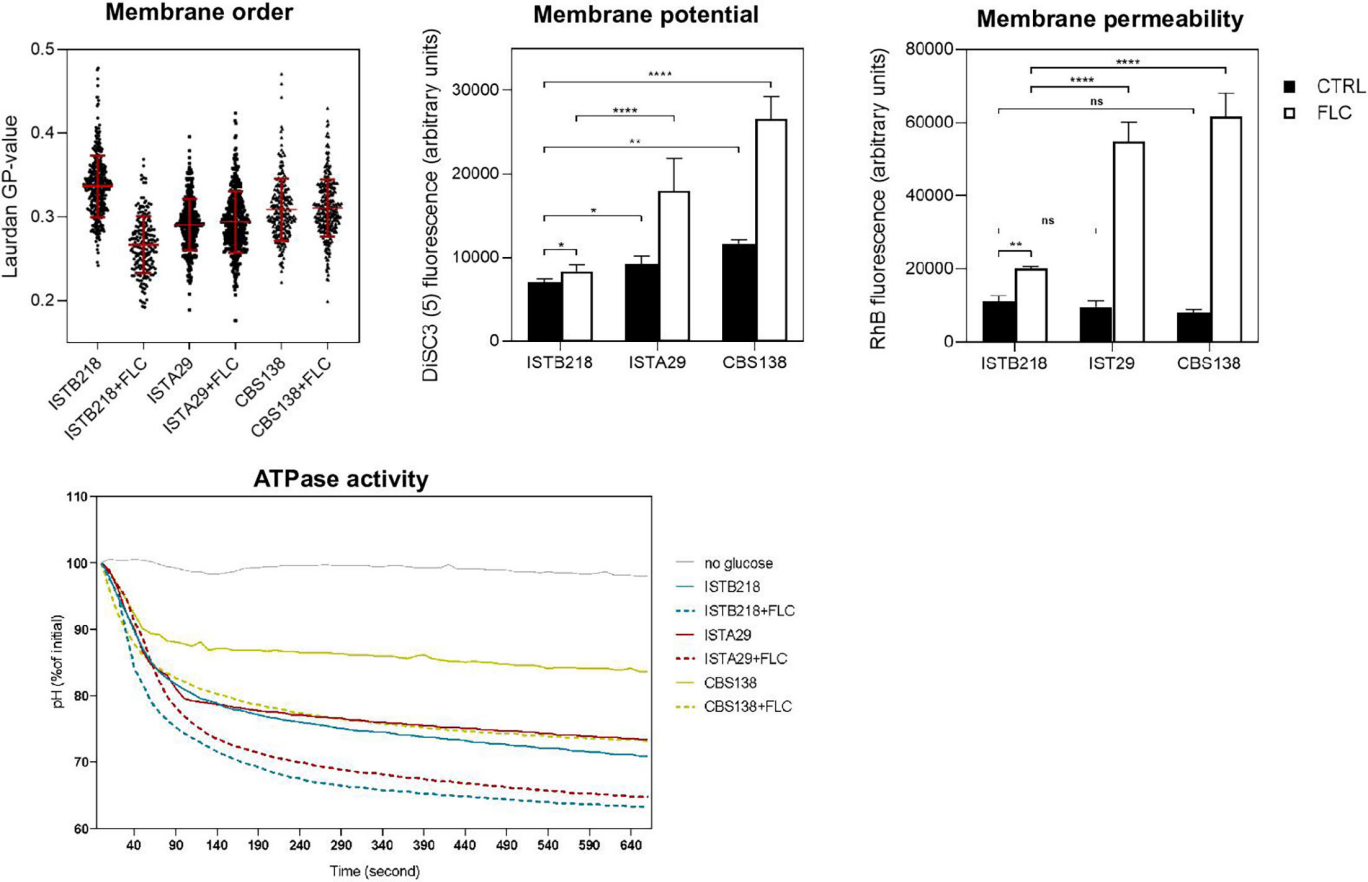
Biophysical properties (order, electrochemical potential, and permeability) of the plasma membrane and activity of the proton pump CgPma1 in the azole-resistant isolate *C. glabrata* ISTB218 and in the azole-susceptible isolates *C. glabrata* ISTA29 and CBS138. (Upper panel, left) Membrane order was estimated based on the calculation of GP values of the fluorescent probe laurdan, estimated in more than 100 individual cells, as detailed in Materials and Methods. (Upper panel, middle) Electrochemical potential was determined upon cell labeling with DiSC₃(5) (3,3′-dipropylthiadicarbocyanine iodide), while membrane permeability (upper panel, right) was estimated upon cell labeling with rhodamine B, as detailed in Materials and Methods. All the data represented are means ± SD of three independent biological replicates. For the statistical analysis, two-way ANOVA followed by Tukey’s *post-hoc* test was used. *P-value below 0.1; **P-value below 0.01; ****P-value below 0.0001; ns, not significant. (Lower panel) Activity of the plasma membrane proton pump in isolates ISTB218, ISTA29, and CBS138, assessed based on whole-cell capacity to acidify the medium upon addition of a glucose pulse to de-energized cells of the different isolates, as detailed in Materials and Methods. The *y*-axis represents the normalized decrease in extracellular pH (as a % of the initial value). The results obtained represent means of three technical replicates obtained from two independent biological replicates.

To assess the extent of fluconazole’s impact over biophysical properties of the membrane in the three strains, we labeled the cells with DiSC_3_, rhodamine B, and laurdan after 24 h of cultivation in the presence or absence of fluconazole (Fig. 4). As observed in the data shown in Fig. 1, fluconazole reduced plasma membrane order of ISTB218 cells (evidenced by the lower average mean of laurdan GP), a response not observed in ISTA29 or CBS138 cells, which maintained identical fluidity levels in the presence or absence of the azole (Fig. 4). Fluconazole-induced dissipation of the plasma membrane potential and increase in permeability of the membrane were also clearly more evident in ISTA29 and CBS138 strains than in ISTB218 (Fig. 4). As the plasma membrane potential in *N. glabratus* (as in most Fungi) is largely dependent on the activity of the plasma membrane proton pump (35), we compared the activity of this pump in ISTB218, CBS138, and ISTA29. A clearly higher activity of Pma1 was observed for ISTB218 and ISTA29, compared to CSB138 (Fig. 4), both in the presence or absence of fluconazole (Fig. 4). Notably, the higher activity of the proton pump in ISTB218 cells is consistent with the higher plasma membrane potential of these cells, as estimated by DiSC_3_ labeling.

### The higher pools of ergosterol detected in ISTB218 depend on the expression of the alternative reductase *CYB5*, which also impacts the azole-resistance phenotype and the biophysical properties of the plasma membrane of this azole-resistant strain

**Fig 5:**
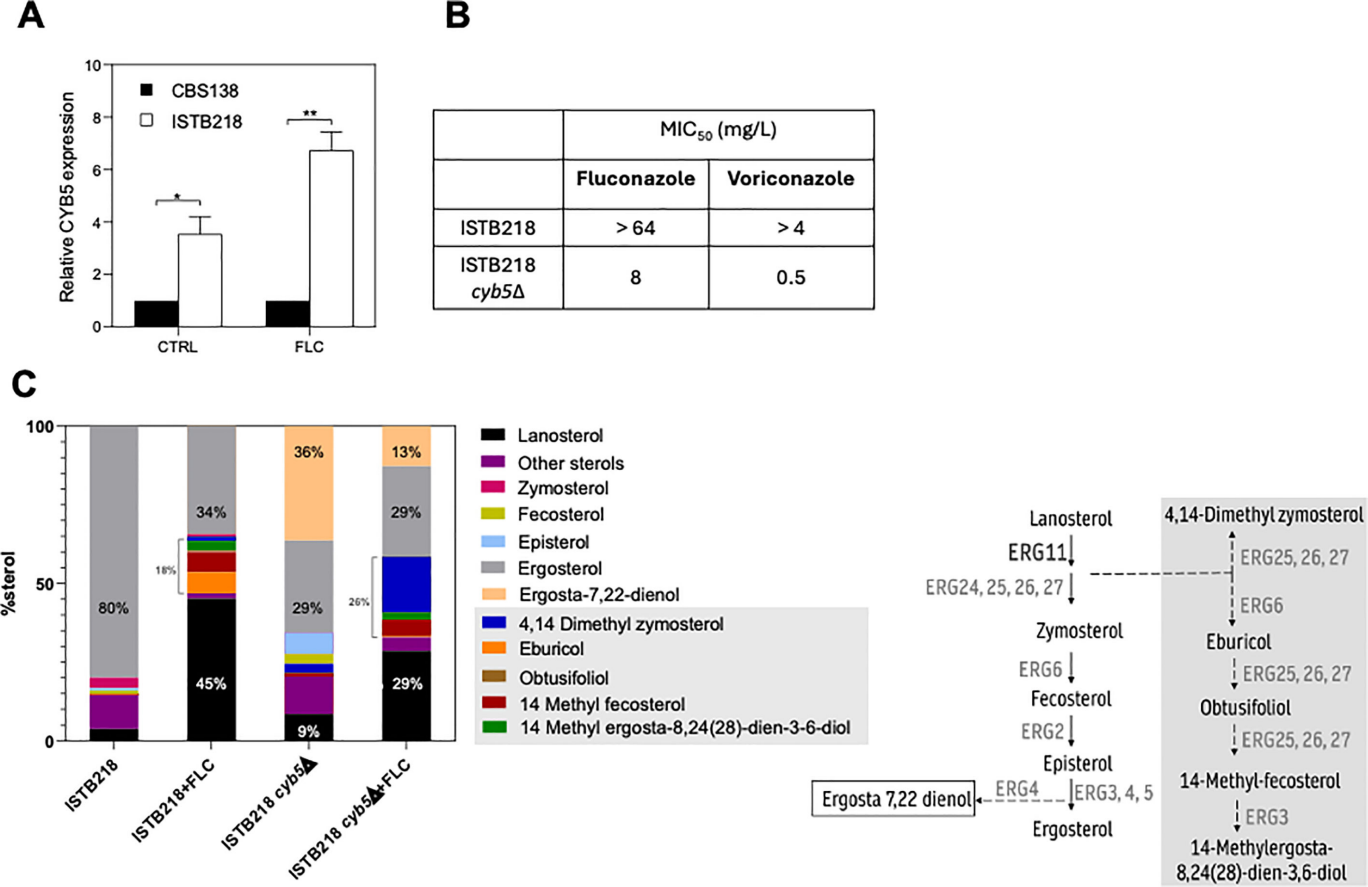
Effect of *CgCYB5* expression in *C. glabrata* azole tolerance and in sterol abundance. (**A**) Transcript levels produced by IST218 or CBS138 cells after cultivation for 24 h in RPMI medium, either supplemented with or without 32 mg/L of fluconazole. CgCYB5 expression was quantified using real-time RT-PCR with ACT1 as an internal control. The results represent the means of in three independent biological replicates. *P-value below 0.1; **P-value below 0.01. (**B**) Minimum inhibitory concentrations (MIC₅₀) of fluconazole and voriconazole in ISTB218 and in ISTB218 Δ*cyb5*, as quantified using the EUCAST-recommended highly standardized growth medium. In Fig. S8, the OD of the cultures obtained in the presence or absence of fluconazole is shown and that resulted in the MICs that are herein presented. The assays were performed in triplicate, yielding essentially similar results. (**C**) Abundance of sterols in ISTB218 and in the derived mutant ISTB218_Δ*cyb5* after cultivation for 24 h in the presence or absence of 32 mg/L fluconazole. The bars represent the relative proportion (% of the total) of each sterol, shown as the mean obtained in three independent biological replicates. The figure highlights the percentage of lanosterol, ergosterol, the total amount of methylated sterols, and ergosta-7,22-dienol. The quantitative data used to build this graph are shown in Fig. S7. On the left of this panel is a simplified scheme of the ergosterol biosynthetic pathway in *C. glabrata*, highlighting the sterols that accumulate in the Δ*cyb5* mutant.

**Fig 6:**
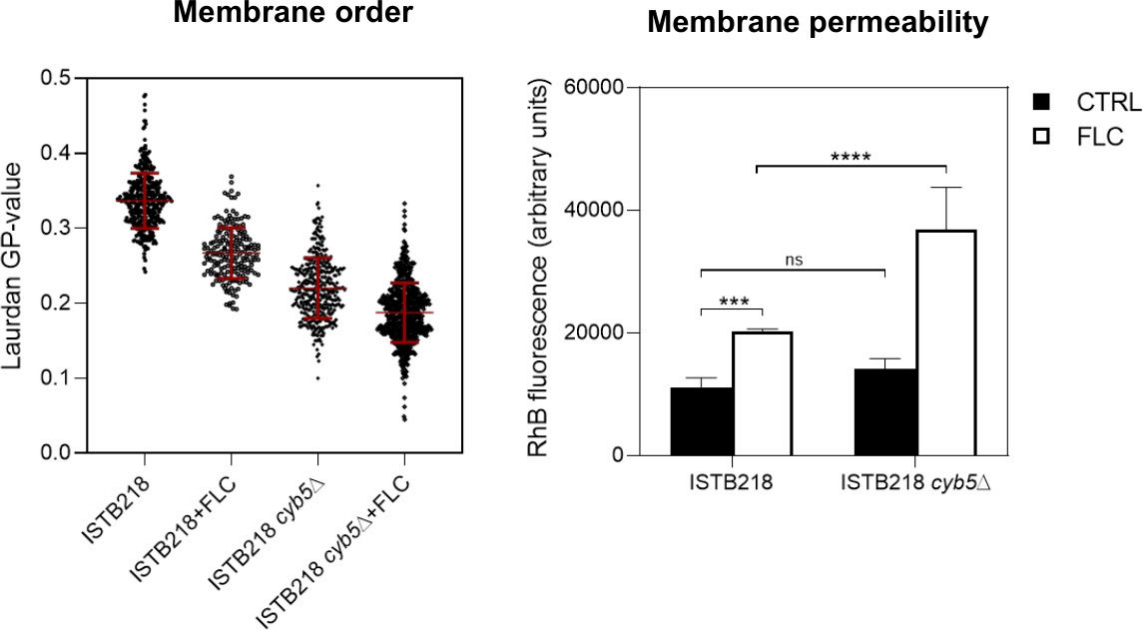
Effect of the expression of *CgCYB5* in plasma membrane order and permeability to rhodamine B of *C. glabrata* ISTB218 cells. ISTB218 cells or ISTB218_Δ*cyb5* cells were cultivated in the presence or absence of fluconazole in the same experimental conditions as those used to quantify MIC. After cultivation, cells of the two isolates were labeled with laurdan, and the GP value was estimated. The values present correspond to the measures made in 100 individual cells. Membrane permeability was estimated upon cell labeling with rhodamine B, as detailed in Materials and Methods. All the data represented are means ± SD of three independent biological replicates. For the statistical analysis, two-way ANOVA followed by Tukey’s *post-hoc* test was used. ***P-value below 0.001; ****P-value below 0.0001; ns, not significant.

The higher internal ergosterol pools detected in ISTB218 cells, along with the reduced accumulation of lanosterol and of methylated sterols upon fluconazole stress, suggested compensatory activity to Erg11 in this azole-resistant strain. In *S. cerevisiae*, it has been described that the alternative electron reductase Cyb5 can serve as a redox partner supplying electrons to Erg11 in the demethylation of lanosterol to zymosterol, usually dependent on Ncp1 (36). Having this in mind, we went back to our previous OMICS data of ISTB218 and CBS138 (13) to search for changes in *CYB5* expression (CAGL0L03828g). Non-stressed ISTB218 cells produced higher transcript levels of *CYB5*, compared to those of CBS138 cells (13); however, due to microarray hybridization problems, we had no data for the cells exposed to fluconazole. Using real-time RT-PCR, we confirmed the microarray results and also found that the transcription of *CYB5* in ISTB218 is even higher under fluconazole stress (Fig. 5A). This overexpression prompted us to delete *CYB5* from the ISTB218 genome, which resulted in a drastic decrease in the strain’s tolerance to fluconazole and voriconazole (Fig. 5B and Fig. S5). Consistent with its anticipated function, ISTB218_Δ*cyb5* mutant cells had lower ergosterol concentrations compared to the original ISTB218 strain (Fig. 5C and Fig. S6). This impact of *CYB5* expression in the ergosterol pool was detectable even in the absence of fluconazole (Fig. 5C and Fig. S6 and S7). Besides reducing ergosterol, deletion of *CYB5* increased episterol and the alternative dienol ergosta-7,22-dienol (Fig. 5C and Fig. S8), supporting the idea that it serves as an electron supplier not only to lanosterol demethylation but also for the conversion of episterol to ergosterol (Fig. 5B and Fig. S5). Similar observations were made for ScCyb5 and CaCyb5 (36–38). Consistent with the prominent modifications found in sterol profiles, ISTB218_Δ*cyb5* cells exhibited lower membrane order (reflected by lower GP values of laurdan) and higher permeability to rhodamine B, especially evident when the cells were cultivated in the presence of fluconazole (Fig. 6).

## DISCUSSION

In the present work, we advance forward the physiological and molecular characterization of azole-resistant *N. glabratus* clinical strains that do not encode Pdr1 GOF variants, thereby advancing knowledge about mechanisms impacting the resistance phenotype that are not regulated by this crucial activator. In this effort, we scrutinized the involvement of the Aus1 and Pdh1 pumps in ISTB218 azole resistance. This was driven by the higher expression of these genes in ISTB218 compared to cells of the reference strain CBS138, as well as their reported involvement in azole tolerance in laboratory strains (16, 23). Individual deletion of *AUS1* or *PDH1* abolished the azole-resistance phenotype of ISTB218 to fluconazole and voriconazole, with the MICs of the mutants going well below the resistance breakpoints of 32 mg/L for fluconazole and 8 mg/L for voriconazole. Previous studies have linked azole resistance and upregulation of *PDH1* in *N. glabratus* clinical isolates (19, 39); however, to the best of our knowledge, this is the first study suggesting a similar connection for *AUS1*. Examination of the promoter region of *AUS1* and *PDH1* in ISTB218, as well as in the two susceptible strains, ISTA29 and CSB138 (Fig. S9), revealed some differences, but they were common to the two clinical strains (ISTA29 and ISTB218). Thus, it is possible that the overexpression of these genes results from higher activity of an upstream regulator(s) in the ISTB218 strain. Both Pdh1 and Aus1 are targets of the Pdr1-dependent network; however, comparative transcriptomic profiling of ISTB218 and CBS138 cells does not show evidence that Pdr1 would be more active (hyperactive) in the ISTB218 strain (13). This suggests that another transcriptional regulator could be mediating the higher expression levels of *PDH1* and *AUS1* in ISTB218. Indeed, overexpression of *PDH1* in other azole-resistant *N. glabratus* clinical strains not showing features of Pdr1 hyperactivation was described elsewhere (19). More detailed analyses will be required to investigate the regulatory network involved in the control of *AUS1* and *PDH1* genes, which may be responsible for their upregulation in ISTB218 cells. One set of interesting players to consider in this context are the Upc regulators (*N. glabratus* encodes two, *UPC2A* and *UPC2B*, with the latter being overexpressed in ISTB218 cells), as Upc2A binds *in vivo* to *PDH1* and *AUS1* promoters (40). Also, several gene targets of the Upc network were found to be overexpressed in the ISTB218 strain (13). Interestingly, *CYB5*, which is herein demonstrated to have a pivotal role for the azole-resistance phenotype of ISTB218, is also a documented direct target of Upc2B, which is overexpressed in ISTB218 (40).

The mechanism by which ABC transporters contribute to multidrug resistance (MDR) has been a topic of debate, as reviewed, for example, in references 24, 26. Two mechanisms have generally been considered: (i) the transporter promotes the direct extrusion of the drugs; (ii) the contribution of the ABC transporter for MDR does not necessarily involve the direct extrusion of the drug, but rather depends on its role in the uptake/extrusion of a physiological substrate, whose partition between the intra- and extracellular environment impacts drug tolerance (e.g., by changing the electrochemical gradient) (24, 26, 41). Most studies suggest a direct role for Pdh1 in azole efflux (39, 42), and our data also seem to support that since we could not detect effects of this gene expression in sterol abundance nor in order, permeability, or electrochemical potential of the plasma membrane. As for Aus1, its protective role against azoles was previously linked to its role as an exogenous sterols importer (23). However, we detected the protective effect of *AUS1* against fluconazole in RPMI medium not supplemented with any sterol. Also, the deletion of *AUS1* did not alter sterol abundance (either in the presence or absence of fluconazole) but clearly affected plasma membrane order of ISTB218 cells. Together, these evidences suggest that Aus1 should have another physiological substrate(s). Plasma membrane order is tightly linked to lipid packing and depends greatly on the proportion of the different lipids present therein. Since the abundance of sterols is identical in ISTB218 and in the Δ*aus1* mutant, it is tempting to speculate that maybe this other substrate(s) of Aus1 is a phospholipid, similar to what was shown for the *S. cerevisiae* ABC- MDR transporters ScPdr5 or ScPdr10 (24, 43). In this context, it is interesting that ScAus1 was recently found to interact with phosphatidylserine (PS), although the authors could not conclude if PS is a direct substrate of the transporter (44).

The exploration of fluorescent probes to monitor plasma membrane order, permeability, and electrochemical potential of ISTB218 cells, while scrutinizing the biological role of Aus1 and Pdh1 in azole tolerance, prompted us to also examine these properties in the azole-susceptible strains CBS138 and ISTA29. With this, we observed that fluconazole stress markedly reduced membrane fluidity of ISTB218 cells, but not of the other two strains. Fluconazole stress also decreased membrane fluidity in *C. albicans* and *S. cerevisiae*, including in azole-resistant *C. albicans* strains (34, 45). Such reduction can be a deleterious effect of the azole over the plasma membrane structure (e.g., due to the incorporation of the methylated sterols that could alter the membrane’s properties), a reaction triggered by the cells to improve response or a combination of these two things. Our results support the idea that this response works in favor of the azole-resistance phenotype, as the changes in fluidity could not be prompted by the two azole-susceptible strains, but these exhibited higher levels of methylated sterols and much less ergosterol (a membrane rigidifying agent).

Our study also shed light on the differences in metabolism of ergosterol that were previously anticipated for the ISTB218 strain (13). We confirmed that the higher fluorescence produced by filipin-labeled ISTB218 cells correlates with higher pools of ergosterol, a trait that we linked to these cells overexpressing the alternative reductase Cyb5. Erg11 catalyzes the 14-methylation of lanosterol to ergosterol in three cycles, each requiring two electrons from the nicotinamide adenine dinucleotide phosphate (NADPH)-dependent reductase Ncp1 (36). In *S. cerevisiae* and in *C. albicans*, the 14α-demethylation can alternatively be supported by the cytochrome b5/NADH cytochrome reductase Cyb5 (36–38). Our results support that this is also the case in *N. glabratus*. More than elucidating the mechanism of ergosterol synthesis in this yeast, our results also suggest that the overexpression of *CYB5* helps the acquisition of azole resistance in a clinical *N. glabratus* strain, a phenomenon not yet described before in this species nor in *C. albicans. CYB5* expression increased the abundance of ergosterol and reduced the accumulation of methylated sterols upon fluconazole stress in ISTB218 cells, presumably by improving the supply of electrons to a fluconazole-inhibited Erg11. Recently, deletion of *CYB5* was found to increase susceptibility to fluconazole of the laboratory strain *N. glabratus* BG14 (20), and we also found the same when we deleted *CYB5* in the background of KUE100_chr606 (our unpublished results). It is thus clear that the protective role of Cyb5 against azoles in *N. glabratus* is not restricted to the ISTB218 strain. Impairment of the interaction established between Erg11 and Ncp1 was recently demonstrated to strongly increase azole efficacy against *C. albicans* (46). This clearly underlines the relevance of electron suppliers as eventual therapeutic targets. A characterization of the individual contribution of Ncp1 and Cyb5 to the supply of electrons to Erg11 is lacking, but the fact that Cyb5 expression had an impact in ergosterol synthesis even in the absence of fluconazole suggests a constitutive participation for this reductase in *N. glabratus* ergosterol biosynthetic pathway.

**Fig 7:**
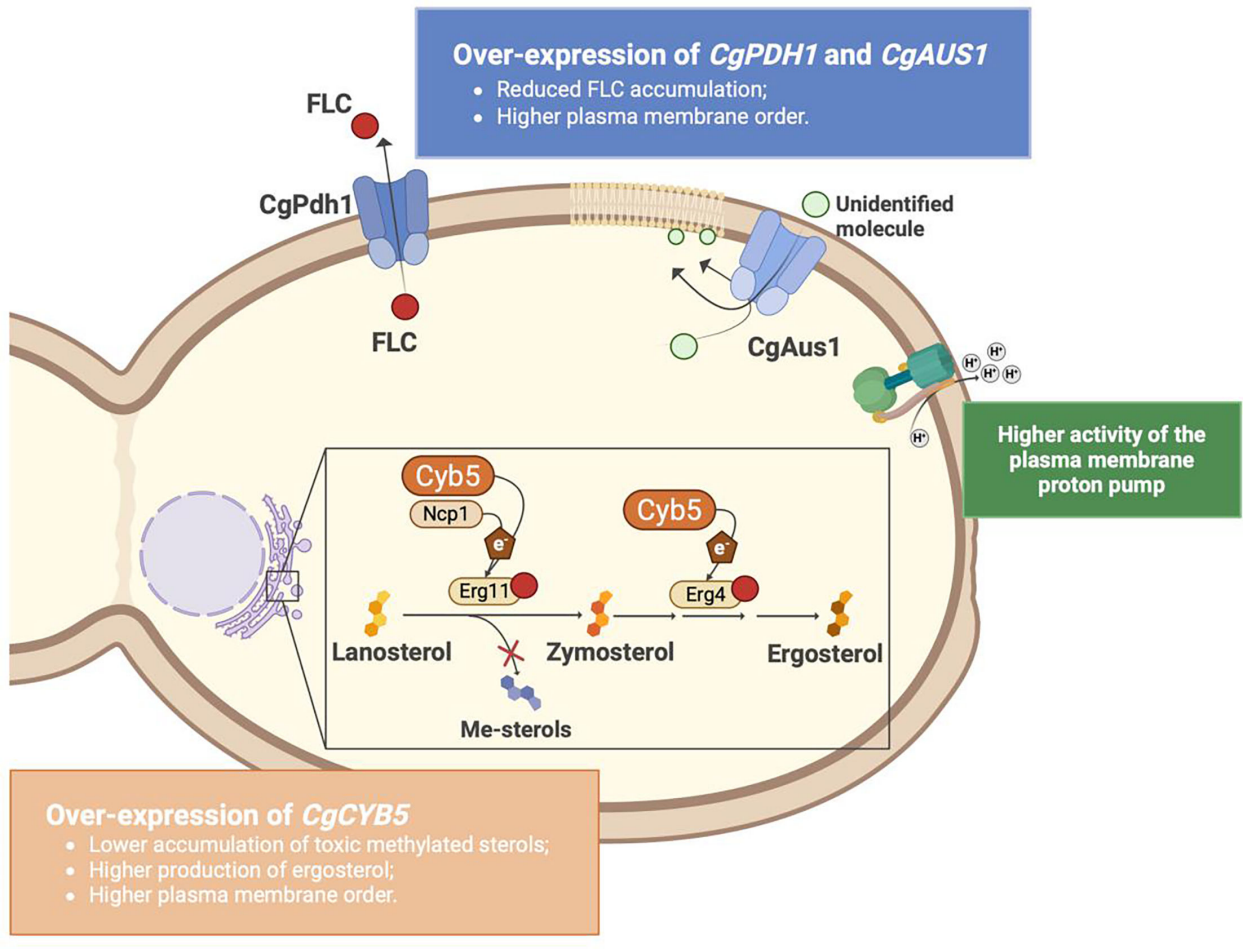
Schematic representation of the involvement of Aus1, Pdh1, and Cyb5 in fluconazole-resistance phenotype of the clinical strain *N. glabratus* ISTB218.

The key findings of this study are summarized in Fig. 7, highlighting the three molecular players that we implicated in the azole-resistance phenotype of the *N. glabratus* ISTB218 strain: Pdh1, Aus1, and the Cyb5 reductase. These findings put these three proteins in the cohort of possible therapeutic targets that could be focused to develop new therapies and treatments that can help bypass the acquisition of resistance in the pathogenic yeast *N. glabratus*.

## MATERIALS AND METHODS

### Strains and growth medium

In this study, we made use of two azole-susceptible strains—the reference clinical strain *N. glabratus* CBS138 and the clinical strain ISTA29 (13)—and 12 azole-resistant strains: ISTB218, ISTE190, ISTD289, ISTD408, ISTE178, ISTE188, ISTE189, ISTF58, FFUL443, FFUL674, FFUL830, and FFUL887, gathered in our laboratory during epidemiological surveys undertaken in Portugal (13, 47). All *N. glabratus* strains were batch-cultured at 30°C, with orbital agitation (250 rpm) in rich growth medium (YPD) or in RPMI1640. YPD contains, per liter, 20 g glucose (Merck Millipore), 10 g yeast extract (HiMedia Laboratories, India), and 20 g peptone (HiMedia Laboratories, India). RPMI1640 contains, per liter, 20.8 g RPMI-1640 synthetic medium (Sigma), 36 g glucose (Merck Millipore), 0.3 g of L-glutamine (Sigma), and 0.165 mol/L of MOPS (3-(N-morpholino)-propanesulfonic acid, Sigma). The pH of the RPMI1640 growth medium was adjusted to 7.0 using NaOH. YPD was sterilized by autoclaving for 15 min at 121°C and 1 atm, while RPMI1640 medium was sterilized by filtration using a 0.22 μm pore size filter. Solid YPD medium was obtained from the formulation used to prepare the corresponding liquid medium, to which 20 g/L agar (Iberagar, Portugal) was added.

### Gene disruption

Deletion mutants devoid of *CYB5*, *AUS1,* and *PDH1* genes were obtained in the background of ISTB218 by replacement with a NAT (Nourseothricin)-resistance cassette. This cassette was amplified by PCR (using primers containing appropriate flanking sequences, available in Table S1) and used to transform ISTB218 cells (using the Alkali-Cation Yeast Transformation Kit [MP Biomedicals] according to the manufacturer’s instructions). The transformed cells were platted onto YPD plates containing 80 mg/L of NAT (Jena Bioscience GmbH), and the successful replacement of each gene by the NAT cassette was confirmed by PCR using specific primers (sequences available in Table S1).

### Azole-susceptibility assays

*N. glabratus* strain ISTB218 and derived mutants ISTB218_*cyb5*∆*,* ISTB218_*upc2b*∆, ISTB218_*aus1*∆*,* and ISTB218_*pdh1∆* were tested for resistance to fluconazole and voriconazole using the EUCAST-recommended standardized microdilution method (48), which allows determination of the MIC_50_ of the two azoles for each strain. Briefly, the different strains were cultivated overnight at 30°C with 250 rpm orbital agitation in YPD. On the next morning, the cells were diluted in 100 μL fresh RPMI (Sigma) to obtain a cell suspension having an OD_600_ of 0.05. These cell suspensions were mixed with 100 μL of fresh RPMI medium (control) or with 100 μL of RPMI medium supplemented with 0.0625–256 mg/L of fluconazole or 0.01–64 mg/L voriconazole. After inoculation, the 96-well plates were incubated without stirring at 37°C for 24 h. After that time, cells were resuspended, and the OD_530_ of the cultures was measured using a microplate reader. The stock solutions of the two azoles were prepared in DMSO (dimethyl sulfoxide, Sigma).

### Quantification of *CYB5* expression

The transcript levels of the *CYB5* gene produced by ISTB218 or CBS138 cells during growth in RPMI medium, supplemented with or without 32 mg/L fluconazole, were quantified by real-time qPCR. For that, the two *N. glabratus* strains were cultivated overnight at 30°C and 250 rpm in 5 mL of YPD. On the following day, cells were re-inoculated (at an OD_600_ of 0.1) in fresh RPMI, either supplemented with or not without 3×2 mg/L fluconazole, and left to grow under the same conditions (30°C, 250 rpm) until mid-exponential phase (OD_600_ ~0.6). At this point, the cells were harvested by centrifugation (8,000 × *g*, 7 min, 4°C; Beckman J2.21, rotor JA.10), and total RNA was isolated using the MasterPure Yeast RNA Purification Kit (Lucigen), following the manufacturer’s instructions. Briefly, cells were resuspended in Proteinase K Extraction Reagent and incubated at 70°C for 15 min, with vortexing every 5 min. After this, samples were cooled on ice for 5 min, and MPC Protein Precipitation Reagent was added. A vortex was applied for 10 s. The debris in the sample was separated by centrifugation, and the supernatant was transferred to a new tube. Isopropanol was added to the supernatant, and RNA was recovered by centrifugation and resuspended in DEPC-treated water. RNA quantification was performed using a Nanodrop set at 260 nm. For the cDNA synthesis step, 1 µg of total RNA from each culture was used in a reverse transcription reaction performed in a C1000 Thermal Cycler (Bio-Rad, Hercules, CA, USA) using the NZY First-Strand cDNA Synthesis Kit (NZYTech). Reagents were mixed according to the manufacturer’s protocol, and the reaction was carried out at 55°C for 30 min, followed by enzyme inactivation at 85°C for 5 min. Samples were then treated with NZY RNase H at 37°C for 20 min to remove residual RNA. The subsequent quantitative PCR step was performed using 2.5 µL of the produced cDNA, primers (cyb5_fw_RT: C GTGACTT TTGACATT and cyb5_rv_RT: CA CTCACTGTTTGGATCA), and SYBR Green Super Mix (BioRad) in a QuantStudio 5 Real-Time PCR System (Applied Biosystems). Gene expression was calculated using gene *ACT1* as an internal control (primers used for *ACT1* amplification were act1_fw_RT: AGAGC TCTTCCCTTCCAT and act1_rv_RT: TTGACCCATAC ACCATG).

### Measures of plasma membrane order, potential, and permeability

The effect of fluconazole on order, electrochemical potential, and permeability of the plasma membrane was measured in ISTB218, ISTB218_Δcyb5, ISTB218_Δaus1, ISTB_Δpdh1, ISTA29, and CBS138 cells cultivated for 24 h at 30°C and 250 rpm in RPMI medium, supplemented with or without 32 mg/L fluconazole (the same experimental setting used for MIC_50_ quantification). At this point, cells were centrifuged (5,000 rpm, 5 min, 4°C), washed twice, and finally resuspended in 1 mL of phosphate-buffered saline buffer (PBS; 137 mM NaCl, 2.7 mM KCl, 10 mM Na₂HPO₄, 1.8 mM KH₂PO₄, pH 7.4, adjusted with HCl). To assess plasma membrane order, the cell suspensions were incubated for 30 min at room temperature, in the dark, with 5 μM of laurdan (Sigma). After this, cells were washed twice with PBS, and then 8 μL of each cell suspension was immobilized on microscope slides (Deltalab) previously prepared using ThermoScientific gene frames and 2.2% agarose (NZYTech). Imaging of the laurdan-labeled cells was acquired using two-photon excitation fluorescence microscopy on a Leica TCS SP5 inverted confocal microscope (DMI600) (Leica Microsystems CMS GmbH, Mannheim, Germany). A Ti:sapphire laser (Mai Tai, Spectra-Physics, Darmstadt, Germany) served as the excitation source, and an apochromatic water immersion objective (63×, NA 1.2; Zeiss, Jena, Germany) was used. The excitation wavelength was set to 780 nm, and laurdan fluorescence emission was collected in two ranges: 400–460 nm and 470–530 nm. GP images were generated and analyzed using custom software developed in MATLAB (Mathworks, Natick, MA). The generalized polarization (GP) value was calculated using the following formula: GP = (I400–460 − G·I470–530)/(I400–460 + G·I470–530). Channel intensities were corrected for background signals, and the calibration factor G was determined by imaging laurdan in DMSO under the same experimental conditions as those applied to the samples. To estimate the plasma membrane potential, the PBS-resuspended cells of the different strains were incubated for 30 min with 10 μM of DiS-C3 (Sigma-Aldrich), in the dark, at room temperature. After this time, fluorescence at 670 nm produced by the different cell suspensions was measured in 96-well plates (Greiner Bio-One) using an SLM-Aminco 8110 Series 2 spectrofluorometer (Rochester, New York, USA). To estimate plasma membrane permeability, the PBS-resuspended cells of the different strains were incubated for 30 min, in the dark, with 100 μM of rhodamine B (acquired from Merck, KGaA, Germany and prepared in a 0.01% ethanol solution). After this time, the cells were observed using fluorescence microscopy (Eclipse 90i, Nikon, Tokyo, Japan), and the excitation and emission wavelengths were 550 nm and 580 nm, respectively.

### Imaging of filipin-labeled *N. glabratus* cells

Cells of *N. glabratus* ISTB218, ISTA29, CBS138, ISTD190, ISTD408, ISTE178, FFUL443, FFUL674, FFUL830, FFUL887, ISTD289, ISTE188, ISTE189, and ISTF58 strains were labeled with filipin III (Sigma) after cultivation in the presence or absence of fluconazole for 24 h under the same experimental setting described above for MIC_50_ quantification. After cultivation, cells were centrifuged (5,000 rpm, 5 min, 4°C), washed twice, and finally resuspended in 1 mL of phosphate-buffered saline buffer (PBS; 137 mM NaCl, 2.7 mM KCl, 10 mM Na₂HPO₄, 1.8 mM KH₂PO₄, pH 7.4, adjusted with HCl). Afterward, the cell suspensions were incubated at room temperature, in the dark, with 15 μM of filipin III and then immobilized in a gene frame. Labeled cells were imaged in a Leica TCS SP5 inverted confocal microscope (DMI600) (Leica Microsystems CMS GmbH, Mannheim, Germany) with an excitation wavelength set to 340–380 nm, and emission being collected between 385 and 470 nm. Images were generated and analyzed using ImageJ software (LOCI, University of Wisconsin).

### Estimation of plasma membrane H^+^-ATPase activity

The activity of the plasma membrane H^+^-ATPase of ISTA29, CBS138, and ISTB218 cells, in the presence or absence of fluconazole, was estimated based on an external acidification assay, as previously described (49). Briefly, cells of the different strains were inoculated in 5 mL of YPD medium and cultivated overnight at 30°C and 250 rpm. On the following day, cells were re-inoculated in fresh RPMI at an OD_600_ of 0.1 and were cultivated under the same conditions until mid-exponential phase (OD_600_~0.6). Cells were harvested by centrifugation (8,000 × *g*, 7 min, 4°C; Beckman J2.21 Centrifuge, rotor JA.10), washed twice with distilled water, and incubated in sorbitol (20 g/L) for 30 min to deactivate plasma membrane H^+^-ATPase. After that time, cells were washed and resuspended in water to obtain a dense cell suspension (OD_600_ of ~20). pH measurements were conducted in a water-jacketed cell with a 10 mL capacity, at 30°C. The chamber contained 3 mL of water (at pH 6.5), 1 mL of the cellular suspension, and 1 mL of glucose (100 g/L). Changes in extracellular pH were monitored immediately after glucose addition and recorded, for 11 min, via potentiometry using a pH microelectrode (Metrohm 6.0204.000) connected to a pH meter (Metrohm 605).

### Sterol quantification

The abundance of sterols in whole cells of strains ISTA29, CBS138, ISTB218, and in ISTB218 *cyb5*∆*,* ISTB218 *aus1*∆*,* and ISTB218 *pdh1*∆ mutants was quantified upon cultivation of these cells in the presence or absence of fluconazole for 24 h (32 mg/L). Briefly, cells were inoculated in 5 mL of YPD medium and cultivated overnight at 30°C with shaking at 250 rpm. On the following day, cells were re-inoculated (at an OD_600_ of 0.1) in fresh RPMI, either supplemented with or without 32 mg/L fluconazole, and cultivated under the same conditions. After 24 h, cells were harvested by centrifugation (8,000 × *g*, 7 min, 4°C) and immediately frozen at −80°C until further use. Non-saponifiable lipids were extracted from cell pellets with hexane after saponification with alcoholic KOH, as previously described (25). Sterols were derivatized using 0.1 mL BSTFA-TMCS (99:1, Sigma) and 0.3 mL anhydrous pyridine (Sigma) by heating at 80°C for 2 h. TMS-derivatized sterols were analyzed using GC/MS (Thermo 1300 GC coupled to a Thermo ISQ mass spectrometer, Thermo Scientific) and identified with reference to relative retention times, mass ions, and fragmentation spectra. GC/MS data files were analyzed using Xcalibur software (Thermo Scientific). Sterol composition was calculated from peak areas as the mean of three replicates.

## SUPPLEMENTAL MATERIAL

Supplemental figures spectrum.03344-25-s0002.pdf Figures S1 to S9.

Table S1 spectrum.03344-25-s0001.pdf List of oligonucleotides used in this study.

ASM does not own the copyrights to Supplemental Material that may be linked to, or accessed through, an article. The authors have granted ASM a non-exclusive, world-wide license to publish the Supplemental Material files. Please contact the corresponding author directly for reuse.
