# Supplementary material for: The azole-resistance phenotype of a Nakaseomyces glabratus clinical strain encoding a wild-type PDR1 allele involves the efflux pumps Aus1 and Pdh1 and Cyb5, an alternative reductase required for ergosterol biosynthesis
Source: Microbiol Spectr. 2026 Mar 27;14(5):e03344-25. doi: 10.1128/spectrum.03344-25 (PMC13141911; doi:10.1128/spectrum.03344-25)
Supplement: Supplemental figures — Figures S1 to S9. [file spectrum.03344-25-s0002.pdf]

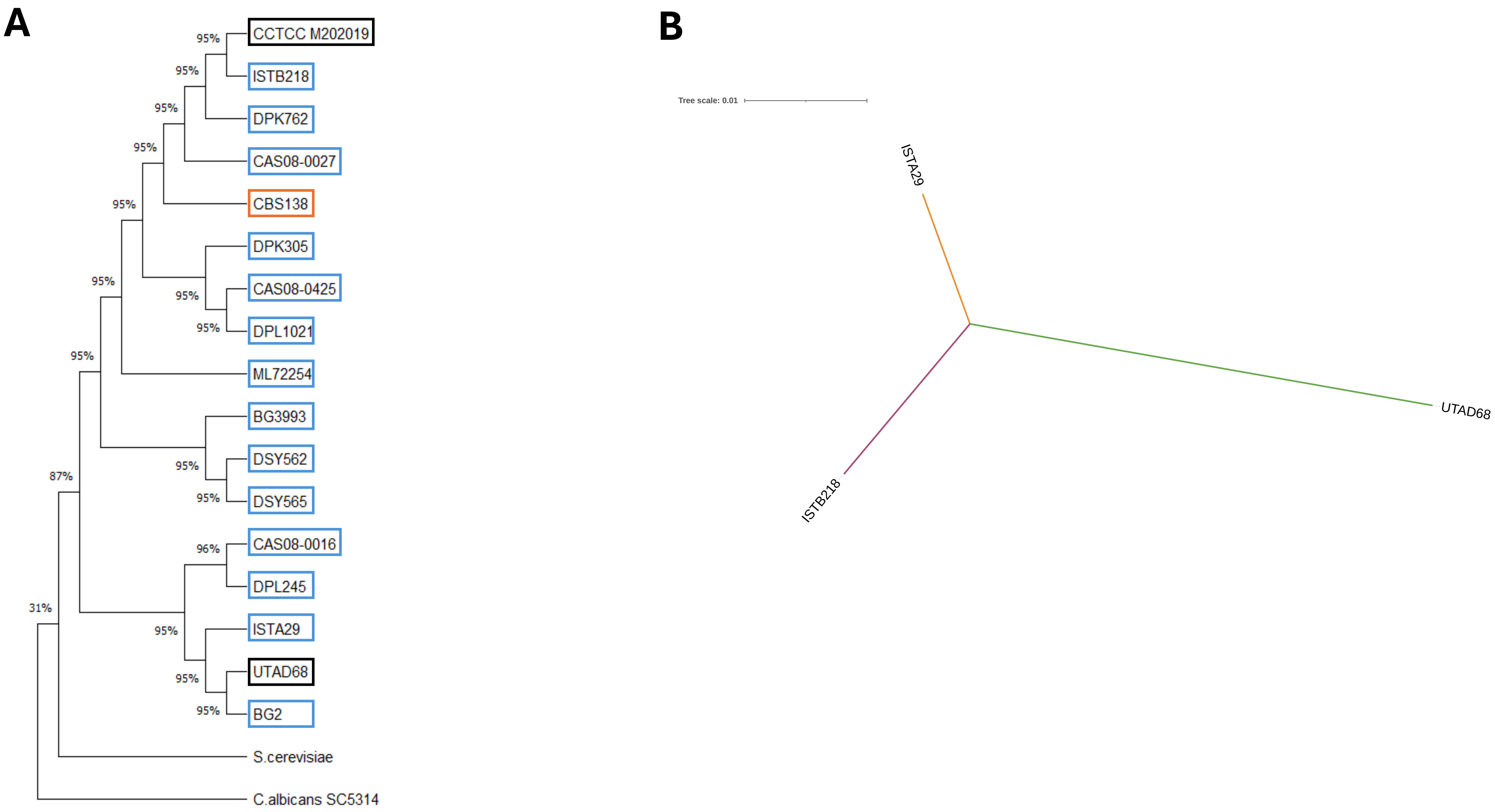
A
CCTCC M202019
ISTB218
DPK762
CAS08-0027
CBS138
DPK305
CAS08-0425
DPL1021
ML72254
BG3993
DSY562
DSY565
CAS08-0016
DPL245
ISTA29
UTAD68
BG2
S.cerevisiae
C.albicans SC5314
95%
96%
87%
31%
B
Tree scale: 0.01
ISTA29
UTAD68
ISTB218

Figure S1: Genetic relatedness of the N. glabratus strains ISTB218, ISTA29 and CBS138, used throughout this work, based on specific gene sequences (A) or using the information obtained from whole-genome sequencing (B). For the phylogenetic tree shown in A, a concatenated two-locus approach to examine genetic relatedness of UTAD68 with the reference strain *C. glabrata* CBS138, the pyruvate-producing *C. glabrata* strain (CCTCC M202019) and 14 clinical strains from environmental (UTAD68, not published) and clinical (ISTB218, ISTA29, DPK762, CAS08-0027, DPK305, CAS08-0425, DPL 1021, DSY562, DSY565, BG3993, ML72254, BG2, CAS08-0016, DPL245) origin. As outgroups, *S. cerevisiae* S288C and *C. albicans* SC5314 were included to root the tree. To build the sequence the gene sequences of *SLG1* and *TRP1* genes were obtained from the NCBI dataset platforms for all strains. For strains *C. glabrata* CBS138 and *C. albicans* SC5314, the gene sequences were obtained from the *Candida* Genome Database (CGD), while for *S. cerevisiae* S288C, sequences these were obtained from the Saccharomyces Genome Database (SGD). The sequences were aligned using the CLUSTALW algorithm within the MEGA software suite v11.0.13 (Mega Cloud Services Limited). The aligned *SLG1* and *TRP1* sequences were then concatenated end-to-end for each strain to generate a single continuous sequence per isolate, increasing the number of phylogenetically informative sites and enhancing tree resolution. A neighbor-joining phylogenetic tree was subsequently constructed using MEGA, employing the default distance model and performing a bootstrap analysis with 1,000 replicates to assess the statistical support of the resulting clades. Arastehfar et al. (doi: 10.1016/j.simyco.2021.100133) demonstrated that the combination of these two loci provides phylogenetic resolution equivalent to that of the full six-locus MLST scheme. The phylogenetic tree shown in B encompasses the SNPs detected in the clinical strains ISTA29 and ISTB218 (whose identification was made previously and detailed in Salazar et al., 2022) compared to the sequence of CBS138 available at *Candida* genome database.

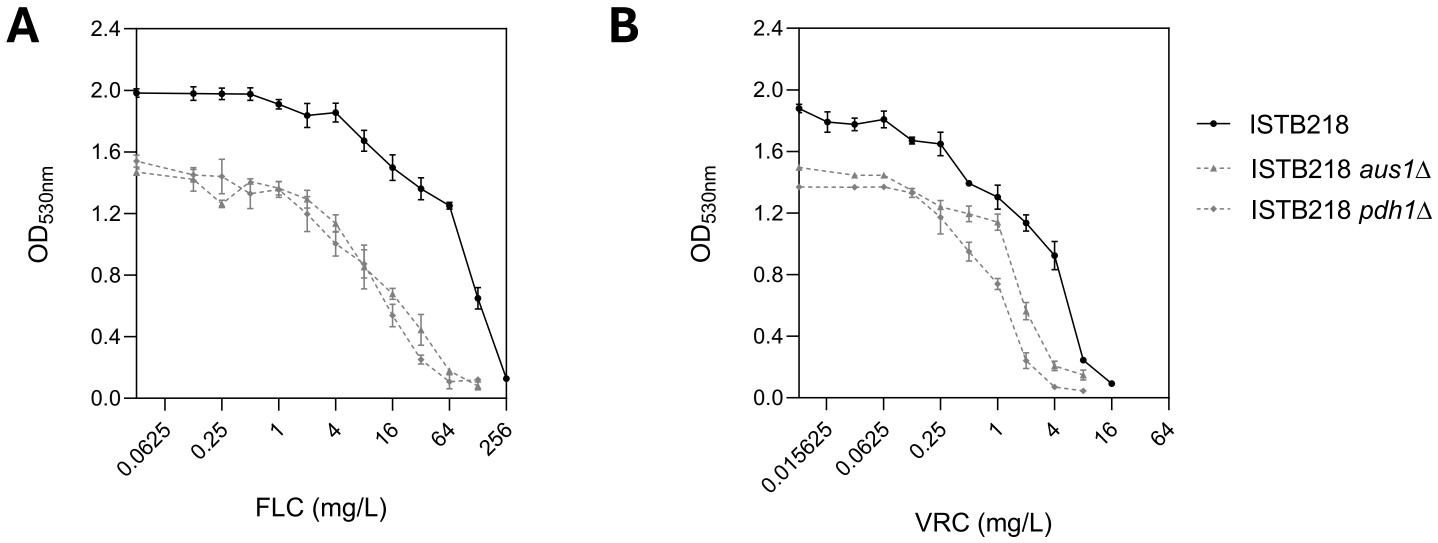

Figure S2: Azole susceptibility of *C. glabrata* ISTB218 strain and of the mutants devoid of *CgPDH1* or *CgAUS1* genes. OD_{600nm} of the cultures of the three strains was registered after 24h of cultivation in RPMI either or not supplemented with increasing concentrations of fluconazole (FLC) or voriconazole (VRC).

Figure S2

	ISTB218	ISTB218+FLC	*aus1*Δ	*aus1*Δ+FLC	*pdh1*Δ	*pdh1*Δ+FLC
**Lanosterol**	3.8	45.2	3.3	43.5	3.5	44.6
**Other sterols**	10.8	1.7	10.6	3.3	12.0	2.0
**Zymosterol**	3.3	0.8	7.2	1.6	5.3	2.2
**Fecosterol**	1.0	-	1.3	-	1.4	-
**Episterol**	0.8	-	0.9	-	0.9	-
**Ergosterol**	80.0	34.4	76.7	39.3	76.9	41.0
**Methylated sterols**	0.3	17.9	-	12.3	-	10.2

Figure S3: Sterol composition (in %) obtained from whole *C. glabrata* cells of isolates ISTB218, IST_Δ*aus1* *and* IST_Δpdh1 cultivated (for 24h) in the presence or absence of fluconazole (FLC). Sterol profiles of the azole-resistant isolate ISTB218 and of the derived deletion mutants *Δaus1* and *Δpdh1Δ* during cultivation in RPMI medium supplemented or not with fluconazole (32 mg/L) using the same experimental setup used for the determination of MICs. The relative proportion (%) of each sterol is shown as the mean of the values obtained in three independent biological replicas. The amount of methylated sterols 4,14-dimethylzymosterol, eburicol, obtusifoliol, 14-methyl fecosterol, and 14-methyl ergosta-8,24(28)-dien-3-6-diol was combined in “methylated sterols”.

Figure S3

Figure S4

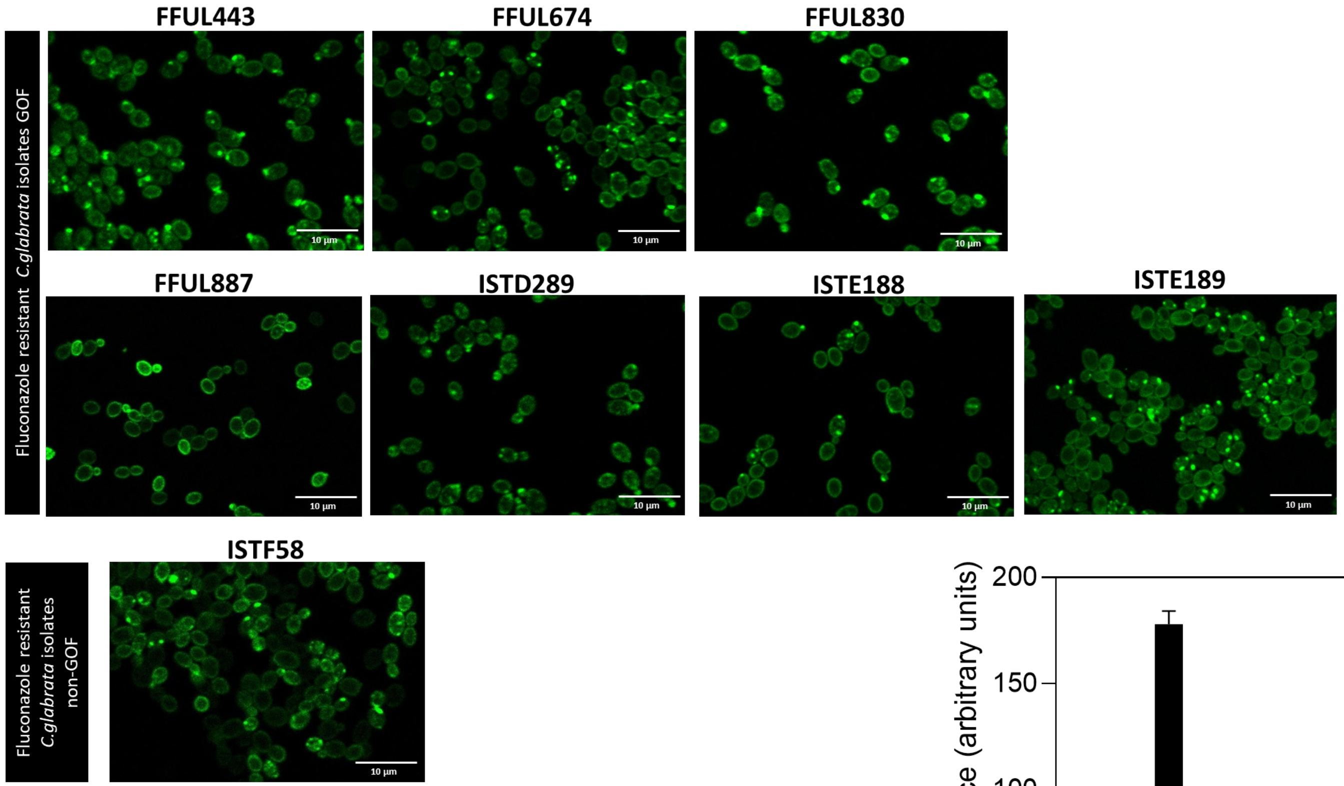

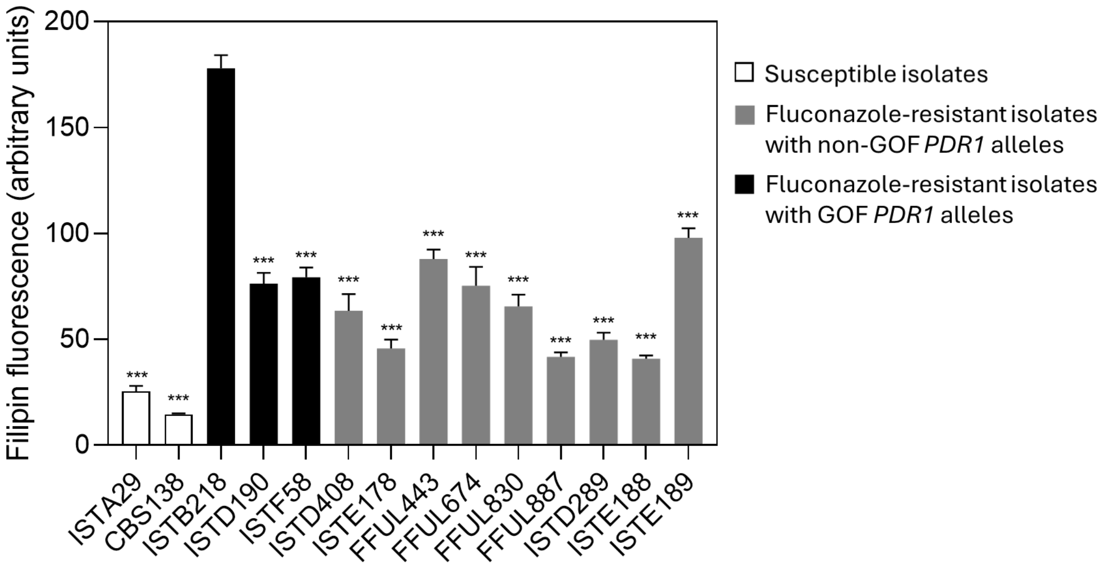

Fig. S4. Filipin staining of *C. glabrata* clinical isolates susceptible or resistant to fluconazole. *C. glabrata* clinical isolates resistant to fluconazole (ISTB218, ISTD190, STD408, ISTE178) were labelled with the ergosterol-binding dye filipin during cultivation in RPMI medium and following the experimental setting described in materials and methods. Scale bar = 10 µm.

	ISTB218	ISTB218+ FLC	ISTA29	ISTA29 +FLC	CBS138	CBS138 +FLC
**Lanosterol**	3.8	45.2	6.2	57.2	7.3	54.4
**Other sterols**	10.7	1.6	11.1	0.7	7.0	2.0
**Zymosterol**	3.3	0.8	1.3	1.0	3.7	1.6
**Fecosterol**	1.0	-	0.4	-	1.2	-
**Episterol**	0.8	-	0.9	-	0.7	-
**Ergosterol**	80.0	34.4	79.8	21.5	79.5	22.8
**Methylated sterols**	0.4	18.0	0.3	19.6	0.6	19.2

Figure S5: Sterol profile of whole-*C. glabrata* cells from isolates ISTB218 (azole-resistant), ISTA29 and CBS138 (azole-susceptible) during cultivation for 24h the presence or absence of fluconazole. Using the experimental setup described in materials and methods, cells from the different isolates cultivated for 24h in RPMI either or not supplemented with 32 mg/L of fluconazole were harvested and their lipid fraction recovered. The figure shows the abundance of different sterols collected in those lipid fractions (in % of the total), shown as the mean of three independent biological replicates. The amount of methylated sterols 4,14-dimethylzymosterol, eburicol, obtusifoliol, 14-methyl fecosterol, and 14-methyl ergosta-8,24(28)-dien-3-6-diol was combined in "methylated sterols".

Figure S5

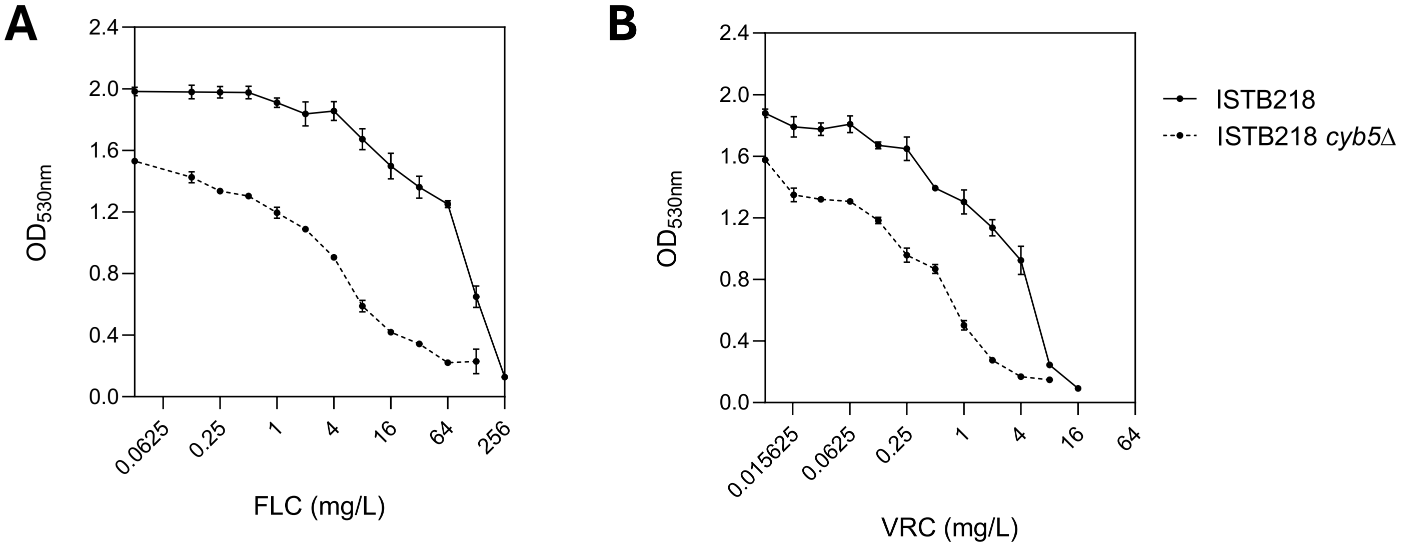

Figure S6: Azole susceptibility of *C. glabrata* ISTB218 strain and of the mutant devoid of *CgCYB5*. OD_{600nm} of the cultures of the two strains was registered after 24h of cultivation in RPMI either or not supplemented with increasing concentrations of fluconazole (FLC) or voriconazole (VRC).

Figure S6

	ISTB218	ISTB218+ FLC	*cyb5*Δ	*cyb5*Δ +FLC
**Lanosterol**	3.8	45.2	8.5	28.6
**Other sterols**	10.7	1.6	11.9	4.3
**Zymosterol**	3.3	0.8	-	-
**Fecosterol**	1.0	-	3.1	-
**Episterol**	0.8	-	6.8	-
**Ergosterol**	80.0	34.4	29.3	28.8
**Ergosta-7,22-dienol**	-	-	36.3	12.7
**Methylated sterols**	0.4	18.0	4.1	25.6

Figure S7. Sterol composition (in %) obtained from whole *C. glabrata* cells of isolate ISTB218 and ISTB218_Δ*cyb5* cultivated (for 24h) in the presence or absence of fluconazole (FLC).

Sterol profiles of the azole-resistant isolate ISTB218 and of the derived deletion mutant *Δcyb5* cultivated for 24h in RPMI medium supplemented or not with fluconazole (32 mg/L) using the same experimental setup used for the determination of MICs. The relative proportion (%) of each sterol is shown as the mean of the values obtained in three independent biological replicas. The amount of methylated sterols 4,14-dimethylzymosterol, eburicol, obtusifoliol, 14-methyl fecosterol, and 14-methyl ergosta-8,24(28)-dien-3-6-diol was combined in "methylated sterols".

Figure S7

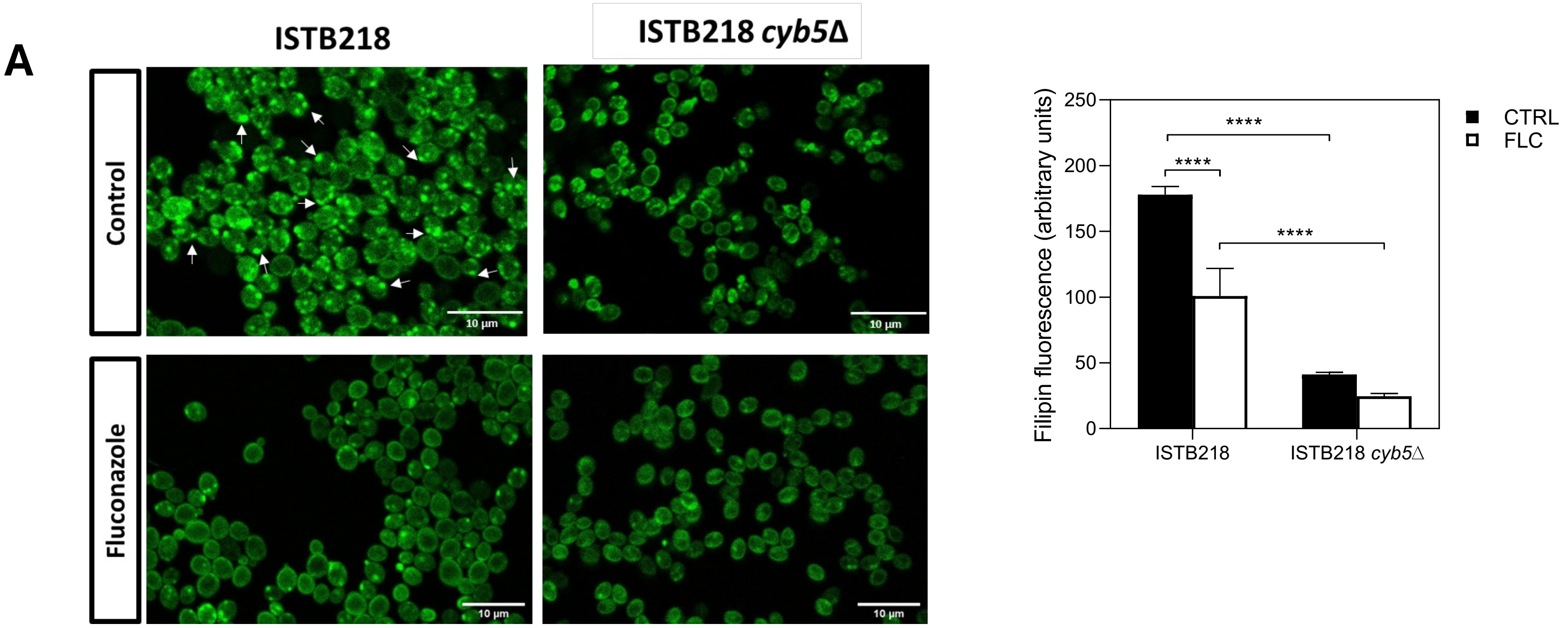

Figure S8: Filipin staining of *C. glabrata* ISTB218 and of the derived deletion mutant ISTB218_Δ*cyb5*. Cells of the azole-resistant isolate ISTB218 or from the mutant devoid of the CgCYB5 gene were labelled with filipin (as detailed in materials and methods) and subsequently imaged in a confocal microscopy where the fluorescence levels produced by whole-cells were gathered. Scale bar = 10 μm.

Figure S8

PDH1 promoter region

```
PDH1_ISTA29      AGACACAGAGAACAGTCTATGGGACCACAAGTTTGCACAAGAGCTGAACAAGTACCGTGA  60
PDH1_CBS138      AGACACAGAAAACAGTCTATGGGACCACAAGTTTGCACAAGAGCTGAACAAGTACCGTGA  60
PDH1_ISTB218     AGACACAGAGAACAGTCTATGGGACCACAAGTTTGCACAAGAGCTGAACAAGTACCGTGA  60
                 *********.**************************************************

PDH1_ISTA29      AGAAACGATGATGAAGCTGGGCAGCGACAAGCTGAAGCCCCACAAGGCGTTTGACGGTGC  120
PDH1_CBS138      AGAAACGATGATGAAGCTGGGCAGCGACAAGCTGAAGCCCCACAAGGCGTTTGACGGTGC  120
PDH1_ISTB218     AGAAACGATGATGAAGCTGGGCAGCGACAAGCTGAAGCCCCACAAGGCGTTTGACGGTGC  120
                 ************************************************************

PDH1_ISTA29      GCTGGGTCCGTTCAACGACAACATGATAGCACAACCGTTCATTCCAAAGCAACTGAAGAG  180
PDH1_CBS138      GCTGGGTCCGTTCAACGACAACATGATAGCACAACCGTTCATTCCAAAACAACTGAAGAG  180
PDH1_ISTB218     GCTGGGTCCGTTCAACGACAACATGATAGCACAACCGTTCATTCCAAAGCAACTGAAGAG  180
                 ************************************************.***********

PDH1_ISTA29      GCGGATGTTGAACCAGAAACCACAAGACCCAGACTACTTAAAGATAAACCAGTTCTTGTA  240
PDH1_CBS138      GCGGATGTTGAACCAGAAACCACAAGACCCAGACTACTTAAAGATAAACCAGTTCTTgta  240
PDH1_ISTB218     GCGGATGTTGAACCAGAAACCACAAGACCCAGACTACTTAAAGATAAACCAGTTCTTGTA  240
                 ************************************************************

PDH1_ISTA29      AATAAATTTGAACTATCCCAACACCAGAGCCCCTTGTATAAATGTAAATACTCAAGAAAT  300
PDH1_CBS138      aataaatttgaaCTATCCCAATACCAGAGCCCCTTGTATAAATGTAAATActcaagaaat  300
PDH1_ISTB218     AATAAATTTGAACTATCCCAACACCAGAGCCCCTTGTATAAATGTAAATACTCAAGAAAT  300
                 ********************* **************************************

PDH1_ISTA29      TAAAGACGTCATTTCAAAAACGCTGATCTACGCTAATTAATATATTATATTAACACATTT  360
PDH1_CBS138      taaagaCGTCATTTCAAAAACGCTGATCTACgctaattaatatattatattaacaCATTT  360
PDH1_ISTB218     TAAAGACGTCATTTCAAAAACGCTGATCTACGCTAATTAATATATTATATTAACACATTT  360
                 ************************************************************

PDH1_ISTA29      TCCAGGCTTCTTATAATTTAATTAGTATTATTGCACAGCAGCAACGAAGTATCCCTATAG  420
PDH1_CBS138      TCCAGGCTTCTTATAATTTAATTAGTATTATTGCACAGCAGCAACGGAGTATCCCTATAG  420
PDH1_ISTB218     TCCAGGCTTCTTATAATTTAATTAGTATTATTGCACAGCAGCAACGAAGTATCCCTATAG  420
                 **********************************************.*************
```

Figure S9

```
PDH1_ISTA29        ATCAACCACGGAATAGTGGATCTTCCGTGGAATATCCATCACAACACTGCACACAGAATT   480
PDH1_CBS138        ATCAACCACGGAATAGTGGATCTTCCGTGGAATATCCATCACAACACTGCACACAGAATT   480
PDH1_ISTB218       ATCAACCACGGAATAGTGGATCTTCCGTGGAATATCCATCACAACACTGCACACAGAATT   480
                   ************************************************************

PDH1_ISTA29        CCACGGAAACCATTGCCTGTTTTGCACTTTTGCACTTTGCACTCTGCACTTTGCACTCTG   540
PDH1_CBS138        CCACGGAAACCATTGCCTGTTTTGCACTTTTGCACTTTGCACTCTGCACTTTGCACTCTG   540
PDH1_ISTB218       CCACGGAAACCATTGCCTGTTTTGCACTTTTGCACTTTGCACTCTGCACTTTGCACTCTG   540
                   ************************************************************

PDH1_ISTA29        CATTAGAGGAACGTATTAACGTTGGTTTCTCACCCACAACCTCGAGGTCTACTAAGGGGT   600
PDH1_CBS138        CATTTGAGGAACGTATTAACGTTGGTTTCTCACCCACAACCTCGAGGTCTACTAAGGGGT   600
PDH1_ISTB218       CATTAGAGGAACGTATTAACGTTGGTTTCTCACCCACAACCTCGAGGTCTACTAAGGGGT   600
                   ****:*******************************************************

PDH1_ISTA29        GGCATAGTCTTCCCCCCCCCACACTATTTATATGCCTAGACAATGGCGTACAGTCAGCAT   660
PDH1_CBS138        GGCATAGTCTC---CCCCCCACACTATTTATATGCCTAGACAATGGCGTACAGTCAGCAT   657
PDH1_ISTB218       GGCATAGTCTTCCCCCCCCCACACTATTTATATGCCTAGACAATGGCGTACAGTCAGCAT   660
                   **********    **********************************************

PDH1_ISTA29        ACCACACGGAGCCGTTAGGAACTTTTTTCTTTTTCATTGTTTTGGCTCTGTGGGAAGAAG   720
PDH1_CBS138        ACCTCACGGAGCCGTTAGgaactttttttctttttcattgttttgGCTCTGTGGGAAGAAG   717
PDH1_ISTB218       ACCACACGGAGCCGTTAGGAACTTTTTTCTTTTTCATTGTTTTGGCTCTGTGGGAAGAAG   720
                   ***:********************************************************

PDH1_ISTA29        GGGGGCATCACAGCATTCCGGGGGGGATGGTCGCCCCATTGCTGTAGTGTGTGCTGCCAG   780
PDH1_CBS138        GGGGGCATCACAGCATTCCGGGGGGGATGGCCGCCCCATTGCTGTAGTGTGTGCTGCCAG   777
PDH1_ISTB218       GGGGGCATCACAGCATTCCGGGGGGGATGGTCGCCCCATTGCTGTAGTGTGTGCTGCCAG   780
                   ****************************** *****************************

PDH1_ISTA29        GAAGGCATTGTTGTGTCTTTAAGACCTCTATCTTGCACTTCACCCCCCCCCTTGCAACAG   840
PDH1_CBS138        GAAGGCATTGTTCTGTCTTTAAGACCTCTATCTTGCACTTCACCCC-CCCCTTGCAACAG   836
PDH1_ISTB218       GAAGGCATTGTTGTGTCTTTAAGACCTCTATCTTGCACTTCACCCCCCCCCTTGCAACAG   840
                   ************ ********************************* *************

PDH1_ISTA29        CCCTCAAAAACTATATTAGCTACTACGTCAATGGCAATTGGTATATATACTGGAACGTTT   900
PDH1_CBS138        CCCTCAAAAACTATATTAGCTACTACGTCAATGGCAATTGGTATATATACTGGAACGTTT   896
PDH1_ISTB218       CCCTCAAAAACTATATTAGCTACTACGTCAATGGCAATTGGTATATATACTGGAACGTTT   900
                   ************************************************************
```

Figure S9

```
PDH1_ISTA29      ATAAGGTTTGTTGGGTGTATTAGGTTGGGAAGTAGGGAACACCACTCAGCTCACCTCAGT   960
PDH1_CBS138      ATAAGGTTTGTTGGGTGTATTAGGTTGGGAAGTAGGGAACACCACTCAGCTCACCTCAGt   956
PDH1_ISTB218     ATAAGGTTTGTTGGGTGTATTAGGTTGGGAAGTAGGGAACACCACTCAGCTCACCTCAGT   960
                 ************************************************************

PDH1_ISTA29      AAAACAAATTATTACAACATAACAAATAGCAAAATAATATAGTGATGAACACACCCGATG   1020
PDH1_CBS138      aaaacaaattattacAACATAACAAATagcaaaataatatagtGATGAACACACCCGATG   1016
PDH1_ISTB218     AAAACAAATTATTACAACATAACAAATAGCAAAATAATATAGTGATGAACACACCCGATG   1020
                 ************************************************************
```

Start codon

Figure S9

AUS1 promoter region

```
AUS1_ISTB218      TGAGATCTGAGATGCGCATCAGAAAGACATTTCCGTTCCTACGGGGAGTATTTAGGGACT  60
AUS1_CBS138       tGAGATCTGAGATGCGCATCAGAAAGACATTTCCATTCCTACGGGGAATATTTAGGgact  60
AUS1_ISTA29       TGAGATCTGAGATGCGCATCAGAAAGACATTTCCGTTCCTACGGGGAGTATTTAGGGACT  60
                  **********************************.************.************

AUS1_ISTB218      TCTTTTTTTGCCACGTTCGTTTAGTTTCTCCGAGAATTAAGGGTCCTACTCACTATGGGG  120
AUS1_CBS138       tctttttttgccaCGTTCGTTTAGTTTCTCCGAGAATTAAGGGTCCTACTCACTATGGGG  120
AUS1_ISTA29       TCTTTTTTTGCCACGTTCGTTTAGTTTCTCCGAGAATTAAGGGTCCTACTCACTATGGGG  120
                  ************************************************************

AUS1_ISTB218      GGTGTATTCCACTGCAGTTACTTGCATGTTGCCTCTCTCTCTCTC----TGTTAAATCTC  176
AUS1_CBS138       GGTGTATTCCACTGCAGTTACTTGCATGTTgcctctctctctctctgtCTGTTAAATCTC  180
AUS1_ISTA29       GGTGTATTCCACTGCAGTTACTTGCATGTTGCCTCTCTCTCTCTC----TGTTAAATCTC  176
                  *********************************************    ***********

AUS1_ISTB218      CCCCCTCCATTGTTCCATTGTTCGTTTTCTTTCTTTTTCCCCTTCTGACACTTTAACGAT  236
AUS1_CBS138       CCCCCTCCATTGTTCCattgtttgatttctttctttttcccCTTCTGACACTTTAACGAT  240
AUS1_ISTA29       CCCCCTCCATTGTTCCATTGTTCGTTTTCTTTCTTTTTCCCCTTCTGACACTTTAACGAT  236
                  ********************** *:***********************************

AUS1_ISTB218      TGCGTGCCCTGCAGGTAATGTGCCGGTAGGAATGCAAACGAATCCCGTTTCACCATCCCA  296
AUS1_CBS138       TGCGTGCCCTGCAGGTAATGTGCCGGTAGGAATGCAAACGAATCCCGTTTCACCATCCCA  300
AUS1_ISTA29       TGCGTGCCCTGCAGGTAATGTGCCGGTAGGAATGCAAACGAATCCCGTTTCACCATCCCA  296
                  ************************************************************

AUS1_ISTB218      TTCATTAGCCACTACGAGATTCTTCCGCCCCGTACAAGTGTCCTGCCATTCGTAATGATG  356
AUS1_CBS138       TTCATTAGCCACTACGAGATTCTTCCGCCCCGTACAAGTGTCCTGCCATTCGTAATGATG  360
AUS1_ISTA29       TTCATTAGCCACTACGAGATTCTTCCGCCCCGTACAAGTGTCCTGCCATTCGTAATGATG  356
                  ************************************************************

AUS1_ISTB218      ATCGTAATCGTTGTTGTAATGGTAATGGTAATCGTACTTGTAATCGTACTTGTAATCGTA  416
AUS1_CBS138       ATCGTAATCGTTGTTGTAATGGTAATGGTAATCGTACTTGTAATCGTACTTGTAATCGTA  420
AUS1_ISTA29       ATCGTAATCGTTGTTGTAATGGTAATGGTAATCGTACTTGTAATCGTACTTGTAATCGTA  416
                  ************************************************************

AUS1_ISTB218      CTTGTAATGATGTCTTGGTGCCATAAGGATTGACTTATACGTAAGGTATGGGGCTAAAGT  476
AUS1_CBS138       CTTGTAATGATGTCTTGGTGCCATAAGGATTGACTTATACGTAAGGTATGGGGCTAAAGt  480
AUS1_ISTA29       CTTGTAATGATGTCTTGGTGCCATAAGGATTGACTTATACGTAAGGTATGGGGCTAAAGT  476
                  ************************************************************
```

Figure S9

```
AUS1_ISTB218      ATAAAACAAATAAAGTAAAACACGCTTTTGTGTTTTTAATATACTCGTACTGGTGCTTAT  536
AUS1_CBS138       ataaaacaaataaagTAAAACACGCTTTTGTGTTTTTAATATACTCGTACTGGTGCTTAT  540
AUS1_ISTA29       ATAAAACAAATAAAGTAAAACACGCTTTTGTGTTTTTAATATACTCGTACTGGTGCTTAT  536
                  ************************************************************

AUS1_ISTB218      ACCATTCGATGTTATTTATTCTTTTTGGCGATTGCAAACAATAATAATCATAACTGCGGA  596
AUS1_CBS138       ACCATTCGAtgttatttattctttttggcGATTGcaaacaataataatcatAACTGCGGA  600
AUS1_ISTA29       ACCATTCGATGTTATTTATTCTTTTTGGCGATTGCAAACAATAATAATCATAACTGCGGA  596
                  ************************************************************

AUS1_ISTB218      GAGCAACTCGCTATATTCGTTCATCCTTTTCTTTGGAAGAATTATTCAAAGAATTACAAA  656
AUS1_CBS138       GAGCAACTCGCTATATTCGTTCATCCTTTTCTTTGGAAGaattattcaaagaattaCAAA  660
AUS1_ISTA29       GAGCAACTCGCTATATTCGTTCATCCTTTTCTTTGGAAGAATTATTCAAAGAATTACAAA  656
                  ************************************************************

AUS1_ISTB218      GAGAGAGAAGAGATTTATCTGATTTCAATAGCTTTTCATATATTAAGAGAGTACTTATTC  716
AUS1_CBS138       GAGAGAGAAGAGATATATCTGATTTCAATAGCTTTTCATATATTAAGAGAGTACTTATTC  720
AUS1_ISTA29       GAGAGAGAAGAGATTTATCTGATTTCAATAGCTTTTCATATATTAAGAGAGTACTTATTC  716
                  **************:*********************************************

AUS1_ISTB218      TTTTGATCCAGGAAGAACAAAAAAAGAGAAGCTTCCTTTTTAGAATAAAATTTTTTTAAA  776
AUS1_CBS138       TTTTGATCCaggaagaacaaaaaaagagaagctTCCTTTttagaataaaatttttttaaa  780
AUS1_ISTA29       TTTTGATCCAGGAAGAACAAAAAAAGAGAAGCTTCCTTTTTAGAATAAAATTTTTTTAAA  776
                  ************************************************************

AUS1_ISTB218      CTTAACTTGTTGCCGCGCTATAGCCATATACAAGTTCTCCTTTCTTTCCGCATCCCCAC  836
AUS1_CBS138       cttAACTTGTTGCCGCGCTATAGCCATATACAAGTTCTCCTATCTTTCCGCATCCCCAC  840
AUS1_ISTA29       CTTAACTTGTTGCCGCGCTATAGCCATATACAAGTTCTCCTTTCTTTCCGCATCCCCAC  836
                  *****************************************:*****************

AUS1_ISTB218      ACCCCCCCAACTATCAATTTTCTTTAAATCAAGGAAAATCTATTACATTCGCTATTAATC  896
AUS1_CBS138       ATCCCCCCAACTatcaattttctttaaatcaAGGAAAATCTATTACATTCGCTATTAATC  900
AUS1_ISTA29       ACCCCCCCAACTATCAATTTTCTTTAAATCAAGGAAAATCTATTACATTCGCTATTAATC  896
                  * **********************************************************

AUS1_ISTB218      TCTACTATCTTTATCTTAGTTTTTTGAAATTCTCGGAAAGAAACATCAAATCAAAAAATT  956
AUS1_CBS138       TCTACTATCTTTATCTtagtttttgaaattctcGGAAAGAAAcatcaaatcaaaaaatt  960
AUS1_ISTA29       TCTACTATCTTTATCTTAGTTTTTTGAAATTCTCGGAAAGAAACATCAAATCAAAAAATT  956
                  ************************************************************
```

Figure S9

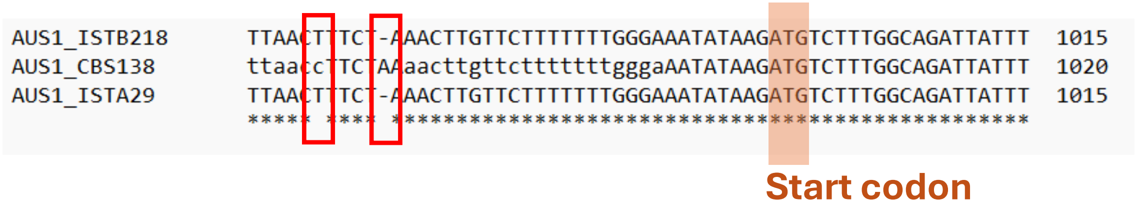

Figure S9. Comparison of the promoter region of genes *AUS1* and *PDH1* in strains *N. glabratus* ISTA29, ISTB218 and CBS138. To get the promoter sequences of the two genes in the clinical strains ISTA29 and ISTB218 the reads obtained after whole-genome sequencing were assembled and the corresponding sequences searched (by BLASTN) for the two coding sequences. The 1000 bp upstream of the ATG start codon were considered the promoter region and were used for a CLUSTALW alignment with the promoter sequence (available at Candida genome database) of the CBS138 strain.

Figure S9
